# Comprehensive analysis and identification of drought-responsive candidate NAC genes in three semi-arid tropics (SAT) legume crops

**DOI:** 10.1186/s12864-021-07602-5

**Published:** 2021-04-21

**Authors:** Sadhana Singh, Himabindu Kudapa, Vanika Garg, Rajeev K. Varshney

**Affiliations:** Center of Excellence in Genomics & Systems Biology, International Crops Research Institute for the Semi-Arid Tropics (ICRISAT), Patancheru, India

**Keywords:** Chickpea, *cis*-acting regulatory elements (CARE), Drought tolerance, Groundnut, Legumes, NACs, Phylogenetics, Pigeonpea

## Abstract

**Background:**

Chickpea, pigeonpea, and groundnut are the primary legume crops of semi-arid tropics (SAT) and their global productivity is severely affected by drought stress. The plant-specific NAC (NAM - no apical meristem, ATAF - *Arabidopsis* transcription activation factor, and CUC - cup-shaped cotyledon) transcription factor family is known to be involved in majority of abiotic stresses, especially in the drought stress tolerance mechanism. Despite the knowledge available regarding NAC function, not much information is available on NAC genes in SAT legume crops.

**Results:**

In this study, genome-wide NAC proteins – 72, 96, and 166 have been identified from the genomes of chickpea, pigeonpea, and groundnut, respectively, and later grouped into 10 clusters in chickpea and pigeonpea, while 12 clusters in groundnut. Phylogeny with well-known stress-responsive NACs in *Arabidopsis thaliana*, *Oryza sativa* (rice), *Medicago truncatula*, and *Glycine max* (soybean) enabled prediction of putative stress-responsive NACs in chickpea (22), pigeonpea (31), and groundnut (33). Transcriptome data revealed putative stress-responsive NACs at various developmental stages that showed differential expression patterns in the different tissues studied. Quantitative real-time PCR (qRT-PCR) was performed to validate the expression patterns of selected stress-responsive, *Ca_NAC* (*Cicer arietinum* - 14)*, Cc_NAC* (*Cajanus cajan* - 15)*,* and *Ah_NAC* (*Arachis hypogaea* - 14) genes using drought-stressed and well-watered root tissues from two contrasting drought-responsive genotypes of each of the three legumes. Based on expression analysis, *Ca_06899, Ca_18090, Ca_22941, Ca_04337, Ca_04069, Ca_04233, Ca_12660, Ca_16379, Ca_16946, and Ca_21186*; *Cc_26125*, *Cc_43030*, *Cc_43785*, *Cc_43786*, *Cc_22429*, and *Cc_22430*; *Ah_ann1.G1V3KR.2*, *Ah_ann1.MI72XM.2*, *Ah_ann1.V0X4SV.1*, *Ah_ann1.FU1JML.2*, and *Ah_ann1.8AKD3R.1* were identified as potential drought stress-responsive candidate genes.

**Conclusion:**

As NAC genes are known to play role in several physiological and biological activities, a more comprehensive study on genome-wide identification and expression analyses of the NAC proteins have been carried out in chickpea, pigeonpea and groundnut. We have identified a total of 21 potential drought-responsive NAC genes in these legumes. These genes displayed correlation between gene expression, transcriptional regulation, and better tolerance against drought. The identified candidate genes, after validation, may serve as a useful resource for molecular breeding for drought tolerance in the SAT legume crops.

**Supplementary Information:**

The online version contains supplementary material available at 10.1186/s12864-021-07602-5.

## Background

Leguminosae, the legume family, is the third-largest family of angiosperms, which is constituted of 800 genera and 20,000 species [1]. Many grain legume crops provide ~ 20–40% of dietary proteins to the world [2]. Among grain legumes, chickpea (*Cicer arietinum*), pigeonpea (*Cajanus cajan*), and peanut or groundnut (*Arachis hypogaea*) are the important food legumes grown predominantly by resource-poor farmers in the semi-arid tropic (SAT) regions of the world. Chickpea, a diploid legume crop species (2n = 2x = 16; genome size of 738.09 Mb), is the second most extensively grown legume with an annual production of ~ 17.19 Mt [3] globally after soybean and provides a rich source of proteins, carbohydrates, vitamins, and minerals for human consumption [4, 5]. Pigeonpea (2n = 2x = 22; genome size of ~ 833 Mb), is another major legume food crop grown on approximately 5 million hectares (ha) with a production of ~ 5.96 Mt annually [3], and is the sixth most important food legume globally. In the developing world, pigeonpea is the primary source of protein to more than a billion people and is the means of sustenance for millions of underprivileged farmers in Asia, Africa, South America, Central America, and the Caribbean [6, 7]. Groundnut, on the other hand, is one of the leading legumes and oilseed crops with high protein content. It is grown widely in the tropics and subtropics with an annual production of ~ 45.95 Mt [3]. Cultivated groundnut (*A. hypogaea* L.) is an allotetraploid (AABB; 2n = 4x = 40; ~ 2.7 Gb genome size), having genome from its diploids ancestors *A. duranensis* (AA) and *A. ipaensis* (BB) [8, 9]. The growth and productivity of these legumes are hugely affected by different biotic and abiotic stresses, which have emphasized the necessity of developing stress tolerant legume cultivars. In the case of chickpea, drought is one of the major constraints which limit crop production [10]. Despite pigeonpea being drought-tolerant and hardy, the crop has limitations under drought stress conditions which lead to yield stagnation. Similarly, groundnut is an oleaginous crop with broad adaptation to tropical and semi-arid climates. However, yield is often compromised when the crop faces water irregularities during the reproductive phase. Furthermore, ominous climate change characterized by enhanced prevalence and severity of drought has spotlighted the adverse impact on plant productivity [11]. Thus, an in-depth understanding of the underlying mechanisms of drought stress tolerance is required to improve the yield potential of these crops.

Over the years, extensive research has been carried out to discover and characterize genes and molecular mechanisms controlling drought responses in both model plants and crops that cope with drought stress conditions [12]. Several transcription factors (TFs) and their DNA binding sites (*cis*-acting regulatory elements), act as molecular switches for stress-responsive altered gene expression, allowing plants to better adapt under adverse conditions [13]. Legumes vary in their response/sensitivity to drought stress. Considering the nutritional and economic benefits, it is important to study the mechanism of drought tolerance in legumes and identify drought-associated genes in these SAT legume crops.

The plant-specific NAC (NAM – no apical meristem, ATAF – *Arabidopsis* transcription activation factor, and CUC – cup-shaped cotyledon) family genes are TFs that constitute one of the largest of plant-specific TF families characterized by a highly conserved NAC domain comprising of approximately 160 amino acid residues at the N-terminus and is further classified into five sub-domains assigned A-E [14]. The N-terminal regions of NAC TFs consist of large number of positively and negatively charged amino acid residues. Sub-domains C and D are rich in basic amino acids and exhibit positive charge. Sub-domains A, C and D are highly conserved domains and are involved in DNA binding attribute. The C-terminus of NAC proteins are variable and can act as either a transcriptional activator or a repressor [15]. The C-terminus region of NAC TFs can also influence oligomerization feature. The NAC TF family was first discovered in *Petunia* more than 22 years ago [16], since then a number of studies have documented the role of NAC genes in a variety of biological processes. For instance, NACs play an important role in lateral root formation [17], seed development [18], leaf senescence [19], stress-inducible flowering induction [20], regulation of secondary cell wall synthesis, cell division [21], plant biotic [22] and abiotic stress responses [23], etc. Furthermore, it was reported that NACs play a significant role in drought stress response and tolerance [24]. Over-expression of *OsSNAC1,* a rice NAC TF, has shown improvement of salt and drought tolerance in wheat cultivars [25]. Similarly, *OsNAC14* caused increased drought resistance in transgenic rice plants by repairing the damaged DNA and defense mechanism [26]. Furthermore, *OsSND2* is known to regulate SCW biosynthesis in rice [27]; *ONAC020*, *ONAC026*, and *ONAC023* genes are involved in seed development [28]; *OsY37* (*Oryza sativa Yellow37*/*ONAC011*) is known to be involved in promoting senescence [29]. *TaNAC29*, a wheat NAC TF, caused improved tolerance against salt and drought [30], while *TaNAC47* displayed enhanced resistance towards PEG, salinity, and freezing stresses in transgenic Arabidopsis plants [31]. *GmNAC109*, a soybean NAC TF, accelerated the formation of lateral roots in transgenic Arabidopsis plants [32]. According to plant transcription factor database (Plant TFDBV4.0) [33], most number of NAC genes reported in plant species are: 138 NAC genes in Arabidopsis (*Arabidopsis thaliana*), 158 in rice (*Oryza sativa*), 189 in maize (*Zea mays*), 165 in foxtail millet (*Setaria italica* L.), 269 in soybean (*Glycine max*), 411 in rapeseed (*Brassica napus*), 289 in poplar (*Populus trichocarpa*), 350 in camelina (*Camelina sativa*), and 200 in eucalyptus (*Eucalyptus grandis*), till now.

In this context, the available draft genome sequences of chickpea [5], pigeonpea [7], and groundnut [8] are important resources and provide an excellent opportunity for a comparative genome survey of novel TFs. Towards this direction, in the present study, comprehensive genome-wide analysis has been performed to identify NAC domain TFs in three SAT legume crops viz., chickpea, pigeonpea, and groundnut. Detailed analyses on their genomic distribution, gene structure, regulatory elements, protein-protein interactions, conserved motifs, and expression patterns under various developmental stages were conducted. As a result, a total of ten, six, and five potential drought-responsive candidate NAC genes were identified in chickpea, pigeonpea and groundnut, respectively. The identified candidate genes serve as valuable resources in the legume breeding program targeting better drought-stress adaptation in these three legume crops.

## Results

### Identification and genomic distribution of NAC proteins/genes in chickpea, pigeonpea, and groundnut

NAC protein sequences from other plant species and NAC Hidden Markov model (HMM) profiles were searched against chickpea [5], pigeonpea [7], and groundnut [8] gene models. Sequences with no apical meristem (NAM) domain were shortlisted. A total of 72, 96, and 166 NAC proteins were identified in genomes of chickpea, pigeonpea and groundnut, respectively (Additional file 1: Table S1). Various physio-chemical properties, such as gene length, protein length, molecular weight (MW), isoelectric point (pI), NAC domain coordinates, and subcellular localization of these genes (Additional file 1: Table S1) were analyzed. The gene length of these NAC genes ranged from 579 bp (*Ca_04309*) to 7259 bp (*Ca_07077*) in chickpea, 170 bp (*Cc_48539*) to 9670 bp (*Cc_22430*) in pigeonpea, and 290 bp (*Ah_ann1.HHSK2A.1*) to 9732 bp (*Ah_ann1.FU1JML.2*) in groundnut. Protein length of these NAC genes varied from 106 AA (*Ca_15515*) to 624 AA (*Ca_14390*), 56 AA (*Cc_48539*) to 627 AA (*Cc_29427*), 62 AA (*Ah_ann1.8AKD3R.1*) to 740 AA (*Ah_ann1.2I3PJC.1*) in the three legumes. Molecular weight of proteins (MW) ranged from 11.86 kDa (*Ca_15515*) to 71.94 kDa (*Ca_14390*), 6.56 kDa (*Cc_48539*) to 71.48 kDa (*Cc_29427*), 7.23 kDa (*Ah_ann1.8AKD3R.1*) to 83.18 kDa (*Ah_ann1.2I3PJC.1*); and isoelectric point (pI) ranged from 4.47 (*Ca_00344*) to 9.6 (*Ca_13012*), 4.5 (*Cc_04140*) to 9.78 (*Cc_22489*), 4.42 (*Ah_ann1.K9ZHT4.1*) to 9.76 (*Ah_ann1.I4FPAQ.1* and *Ah_ann1.W8FFAE.1*) in chickpea, pigeonpea, and groundnut, respectively. Prediction of subcellular localization based on significant similarity in potential location/location DB indicated 73.16 and 64.5% of the identified NAC genes were potentially located in the nucleus of chickpea and pigeonpea, respectively, while in the case of groundnut, only 57.83% of genes were potentially located in the nucleus.

**Fig. 1:**
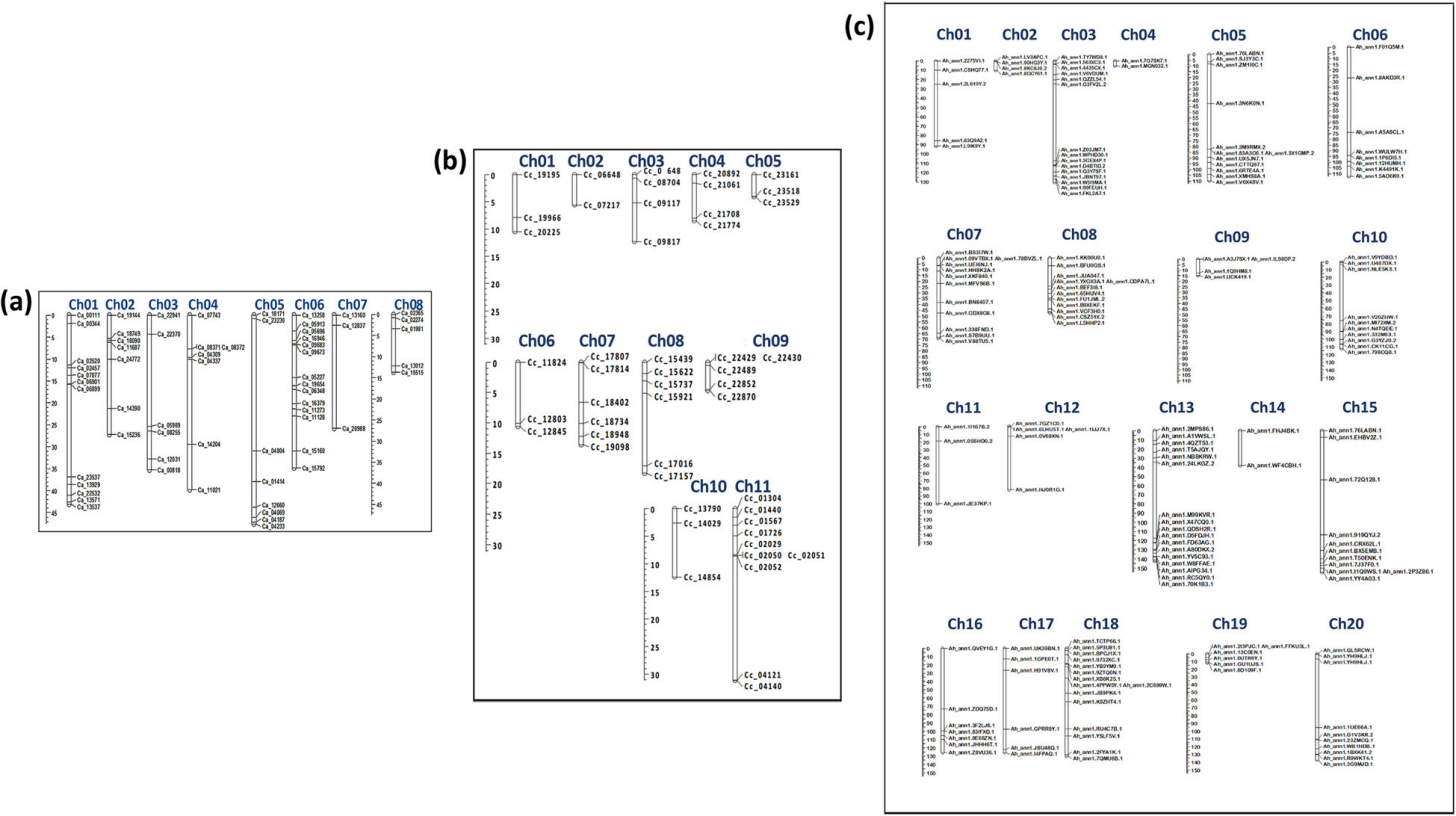
Graphical representation of chromosomal localization of NAC genes in three legume crops using MapChart 2.3.2. **a** Representation of chromosomal localization in chickpea NAC genes. A total of 62 NAC genes are mapped to eight chromosomes (Ch). The exact position of each chickpea NAC genes (*Ca_NAC*) can be estimated using scale on the left (Mbp). **b** Representation of chromosomal localization in pigeonpea NAC genes. A total of 49 NAC genes are distributed among eleven chromosomes (Ch). The position of each pigeonpea NAC gene (*Cc_NAC*) can be estimated using scale on the left (Mbp). **c** Representation of chromosomal localization in groundnut NAC genes. A total of 166 NAC genes are distributed among twenty chromosomes (Ch). The position of each groundnut NAC gene (*Ah_NAC*) can be estimated using scale on the left (Mbp)

A total of 62 out of the 72 (86%) identified NAC genes were distributed across eight chromosomes (Ch01-Ch08) in chickpea (Fig. 1a), 49/96 (51%) were distributed among 11 chromosomes (Ch01-Ch11) in pigeonpea (Fig. 1b), whereas 166/166 (100%) of the NAC genes were located across all 20 chromosomes in groundnut (Fig. 1c). However, 10 genes in chickpea and 47 genes in pigeonpea were anchored on unmapped scaffolds. In chickpea, the minimum number of NAC genes were found distributed on Ch07 (3); whereas the maximum number of NAC genes (14) were identified on Ch06, followed by Ch01 with 12 NAC genes. Similarly, in pigeonpea, only two NAC genes (*Cc_06648,* and *Cc_07217*) were identified on Ch02 and the maximum number of genes (10) was identified on Ch11. However, in the case of groundnut, 17 NAC genes are distributed on Ch13 and 15 genes each on Ch18 and Ch03, followed by 12 genes each on Ch05, Ch07 and Ch08.

### Transmembrane helices and orthologous distribution

Five, eight, and fifteen proteins contain transmembrane helices (TMHs) among the identified 72 *Ca_NACs*, 96 *Cc_NACs*, and 166 *Ah_NACs*, respectively (Table 1). Out of five *Ca_NACs*, four proteins had one TMH except for *Ca_14390*, which contain two TMHs. However, all eight *Cc_NAC* proteins contain one transmembrane domain. In contrast, six *Ah_NAC* proteins (*Ah_ann1.1GPE0T.1, Ah_ann1.A80DKX.2, Ah_ann1.ILS8DP.2, Ah_ann1.JBNT97.1, Ah_ann1.XKF840.1* and *Ah_ann1.FFKU3L.1*) contain two TMHs, while the remaining nine proteins had one TMH.

**Table 1:** Identified putative membrane-bound NAC proteins in chickpea, pigeonpea, and groundnut and predicted number of transmembrane helices (TMHs) using TMHMM v2.0

Gene name	Length (aa)	Number of predicted TMHs	Transmembrane sequences (position)	Expected number, TMHs AAs	Expected number, first 60 AAs
***Chickpea***					
*Ca_14390*	624	2	535–552; 600–622	38.70603	0
*Ca_04337*	558	1	530–552	21.93664	0.00065
*Ca_08372*	577	1	552–574	20.90645	0.0002
*Ca_04069*	612	1	585–607	22.28558	0.00171
*Ca_27204*	610	1	582–604	22.60723	0
***Pigeonpea***					
*Cc_29425*	625	1	533–552	35.48482	0
*Cc_29427*	627	1	535–554	34.48751	0
*Cc_40311*	572	1	548–570	22.73951	0.00529
*Cc_42082*	480	1	456–478	22.28638	0.00195
*Cc_26125*	567	1	538–560	22.04413	0.00047
*Cc_14854*	215	1	151–173	22.67864	0.00049
*Cc_41044*	349	1	326–348	21.84267	0.08532
*Cc_22429*	589	1	564–586	21.13549	0
***Groundnut***					
*Ah_ann1.1GPE0T.1*	678	2	582–604; 656–675	41.84687	0
*Ah_ann1.1IJJ7X.1*	481	1	458–480	19.08509	0.00479
*Ah_ann1.1UE66A.1*	592	1	569–591	22.56847	0.01598
*Ah_ann1.8D109F.1*	499	1	471–493	22.12064	0.00134
*Ah_ann1.8KC8J6.2*	481	1	458–480	19.08496	0.00479
*Ah_ann1.A80DKX.2*	607	2	499–518; 586–605	40.88282	0
*Ah_ann1.CDPA7L.1*	457	1	418–440	22.51853	0.0084
*Ah_ann1.FFKU3L.1*	583	2	531–553; 558–580	40.01905	0
*Ah_ann1.H91V8V.1*	709	1	680–702	22.56598	0.01784
*Ah_ann1.ILS8DP.2*	583	2	531–553; 558–580	39.30286	0
*Ah_ann1.JBNT97.1*	634	2	525–544; 611–633	43.36892	0
*Ah_ann1.MFVS6B.1*	698	1	669–691	22.60288	0.01785
*Ah_ann1.V20ZHW.1*	592	1	569–591	22.56383	0.01596
*Ah_ann1.XKF840.1*	679	2	581–603; 657–676	41.33532	0
*Ah_ann1.BPCJ1X.1*	444	1	406–428	22.49211	0.01105

**Fig. 2:**
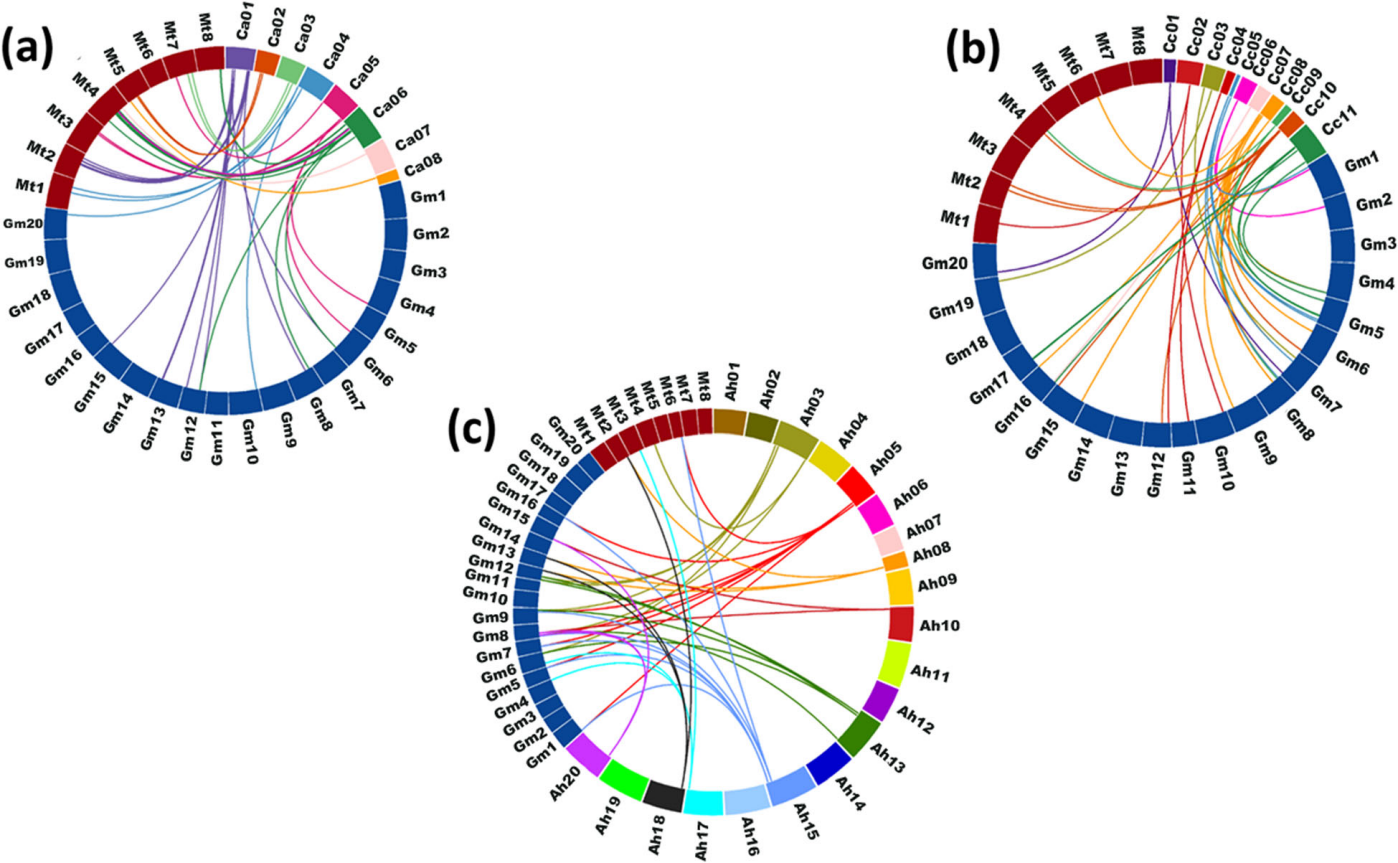
Comparative analysis of orthologous relationship of NAC genes. **a** Chickpea **b** Pigeonpea **c** Groundnut with *Medicago truncatula* and *Glycine max*. Gene orthologs are illustrated using circos [34]. Origin of the strokes represent chromosomal locations of the respective NAC genes, while the strokes represent the orthologous genes of *Medicago truncatula* and *Glycine max*

Two closely-related legumes, *Medicago* and soybean were used to identify orthologs of NAC proteins of chickpea, pigeonpea, and groundnut. Chickpea and groundnut share the maximum orthologs with *Medicago*, whereas pigeonpea with soybean (Fig. 2a, b, c) using parameters mentioned in methodology.

### Phylogenetic relationships and identification of putative stress-responsive NAC genes

**Fig. 3:**
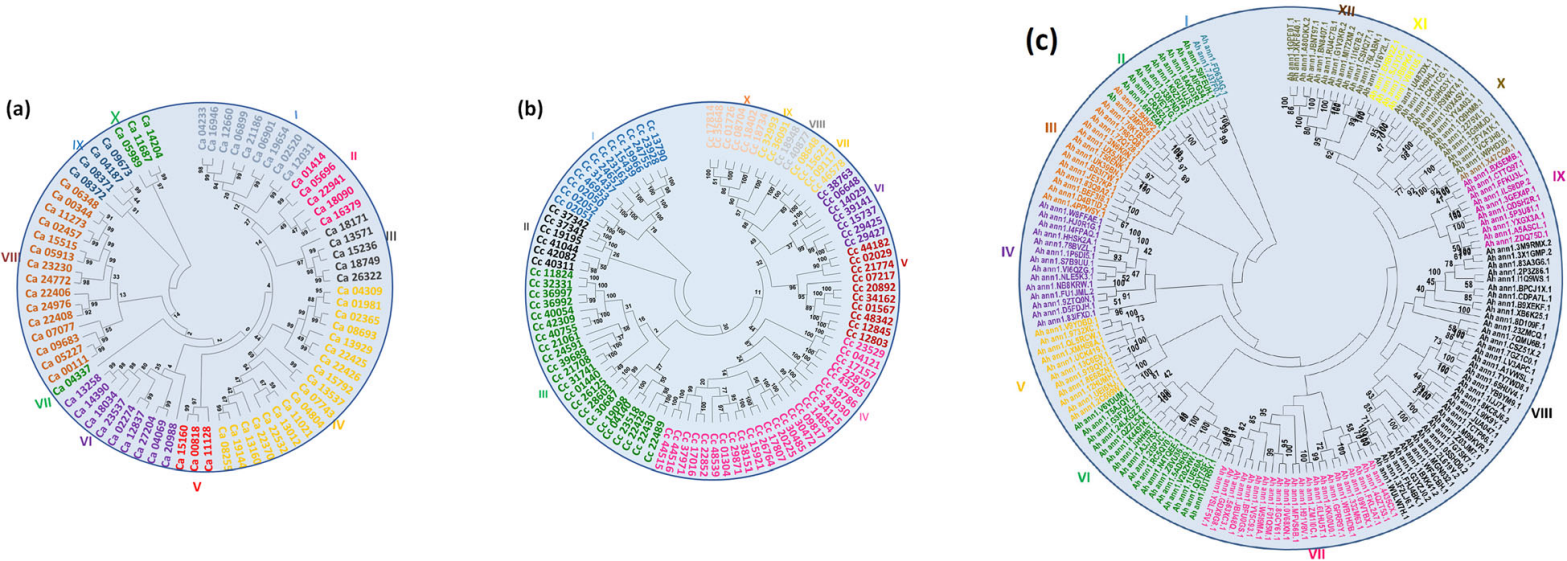
Phylogenetic tree of NAC genes in three legume crops. **a** The phylogenetic tree of NAC genes from chickpea (*Ca_NAC)* was constructed using all 72 protein sequences in MEGA7.0 using the Neighbor-Joining (NJ) method with 1000 bootstrap replicates. Bootstrap values are displayed next to the branch nodes. **b** The phylogenetic tree of NAC genes from pigeonpea (*Cc_NAC*) was constructed using all 96 protein sequences in MEGA7.0 using the Neighbor-Joining (NJ) method with 1000 bootstrap replicates. Bootstrap values are displayed next to the branch nodes. **c** The phylogenetic tree of NAC genes from groundnut (*Ah_NAC)* was constructed using all 166 protein sequences in MEGA7.0 using the Neighbor-Joining (NJ) method with 1000 bootstrap replicates. Bootstrap values are displayed next to the branch nodes

To discover phylogenetic relationships between NAC proteins/genes in chickpea, pigeonpea, and groundnut, an unrooted phylogenetic tree with full NAC protein sequences was constructed. Neighbor-Joining (NJ) method was used with bootstrap values (1000 replicates) (Fig. 3a, b, c). A total of 72, 96, and 166 protein sequences were used. Based on phylogenetic analysis, the *Ca_NACs* and *Cc_NACs* were classified into 10 major groups (Fig. 3a, b) and *Ah_NACs* were classified into 12 broad groups (Fig. 3c). In chickpea, Group IV is the largest clade, with 18 proteins and accounts for 25% of all NAC proteins, followed by group VIII, which has 15 proteins (20.83%). Group VII is the smallest and has only one NAC protein (*Ca_04337*). Groups I and VI contain nine; groups II, and III include five; and groups V, and X have three proteins each. Additionally, groups IV and VIII both contain two subgroups. Likewise, in pigeonpea, group IV is the largest with 25 proteins (26%), followed by group III with 22 proteins (22.9%) which also has different subgroups. Further, groups II and X have six proteins each. In groundnut, among twelve major groups, group VIII is the largest (21.7%) with 36 proteins and group VII contains 22 proteins (13.25%), whereas group I has only two proteins (*Ah_ann1.FD63AG.1* and *Ah_ann1.7J37F0.1*). In addition to this, Groups VI, VII, and VIII also contain two major subgroups (Fig. 3c).

**Fig. 4:**
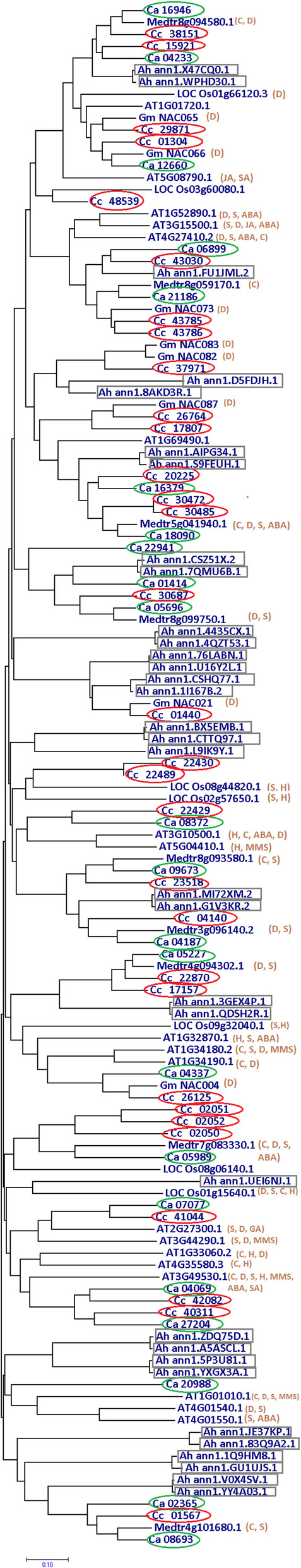
Phylogenetic relationship of putative stress-responsive NAC genes of chickpea (22), pigeonpea (31), and groundnut (33) with well-known stress-responsive NAC genes (43) from *Arabidopsis thaliana*, *Oryza sativa, Medicago truncatula* and *Glycine max* using MEGA7.0. The bar indicates the relative divergence of the sequences examined. Stress-responsiveness of each NAC gene from model crops species is shown next to its name in parentheses. D-dehydration/drought; S-salt stress; C-cold stress; H-heat stress; ABA-abscisic acid; JA-jasmonic acid; SA-salicylic acid; MMS-methyl methane sulfonate

For the prediction of putative stress-responsive NAC genes, a phylogenetic analysis involving complete protein sequences of all identified NAC genes from the three legumes studied and most well-known stress-responsive NAC proteins/genes from *Arabidopsis thaliana*, *Oryza sativa* (rice), *Medicago truncatula* and *Glycine max* (soybean) was conducted. As genes with similar functions are phylogenetically related, 22 abiotic stress-responsive NAC genes/proteins in chickpea, 31 in pigeonpea, and 33 in groundnut were identified (Fig. 4, Additional file 2: Fig. S1, S2, and S3), using known stress-responsive NAC genes from *Arabidopsis* (19 *ANAC*s), *Oryza* (7 *ONACs*), *Medicago* (9 *MtNACs*) and *Glycine max* (8 *GmNACs*). A complete list of the putative stress-responsive genes identified for all the three legumes along with their description is given in Table 2. In addition to this, details regarding the known stress-responsive NACs from the model and crop plants included in the analysis are also provided in Additional file 1: Table S2. As the primary purpose of this study is to discover and explore the potential stress-responsive candidate NAC genes in the three legume crops, further downstream analysis was performed on the putative stress-responsive NAC genes.

**Table 2:** Identified stress-responsive NAC genes/proteins from phylogenetic analysis with known NAC genes (stress-responsive) from model crop species using MEGA 7.0 along with their description and distribution of conserved motifs domains in chickpea, pigeonpea, and groundnut using MEME standalone version 5.0.2

Predicted stress-responsive NAC genes	Description	Total number of motifs	Subdomain (DNA binding NAC domain)
***Chickpea***			
*Ca_06899*	NAC domain-containing protein 72	6	DE
*Ca_21186*	NAC domain-containing protein 72	7	ABCDE
*Ca_12660*	NAC domain-containing protein 2	8	ABCDE
*Ca_04233*	NAC domain-containing protein 2-like	8	ABCDE
*Ca_16946*	NAC domain-containing protein 2-like	8	ABCDE
*Ca_16379*	NAC transcription factor 29-like	7	ABCDE
*Ca_18090*	NAC transcription factor 29	7	ABCDE
*Ca_05696*	NAC transcription factor 25-like	7	ABCDE
*Ca_01414*	NAC transcription factor 29	6	ACDE
*Ca_22941*	NAC transcription factor 25-like	5	ACDE
*Ca_02365*	NAC domain-containing protein 7-like	7	ABCDE
*Ca_08693*	NAC domain-containing protein 7-like	6	ACDE
*Ca_04069*	Uncharacterized protein LOC101492664 isoform X1	6	ABCDE
*Ca_27204*	Protein NTM1-like 9	6	ABCDE
*Ca_20988*	NAC domain-containing protein 69-like isoform X1	5	ABCDE
*Ca_07077*	NAC domain-containing protein 40-like	6	ABCDE
*Ca_08372*	NAC domain-containing protein 53 isoform X2	8	ABCDE
*Ca_04187*	NAC domain-containing protein 78 isoform X1	8	ABCDE
*Ca_09673*	NAC domain-containing protein 78-like	8	ABCDE
*Ca_05227*	NAC transcription factor ONAC010	6	ABDE
*Ca_04337*	NAC domain-containing protein 16-like	7	ABCDE
*Ca_*05989	NAC domain-containing protein 45-like	6	ABDE
***Pigeonpea***			
*Cc_26125*	NAC domain-containing protein 78	7	ABCDE
*Cc_43030*	NAC domain-containing protein 72-like	7	ABCDE
*Cc_43785*	NAC domain-containing protein 72-like	7	ABCDE
*Cc_43786*	NAC domain-containing protein 72-like	7	ABCDE
*Cc_22429*	NAC domain-containing protein 78	7	ABCDE
*Cc_22430*	NAC domain-containing protein 78	6	ABCDE
*Cc_22489*	NAC domain-containing protein 78	7	ABCDE
*Cc_22870*	NAC domain-containing protein 104-like	6	ABCD
*Cc_15921*	NAC domain-containing protein 2-like	7	ABDE
*Cc_17157*	NAC domain-containing protein 104-like	7	ABCDE
*Cc_29871*	NAC domain-containing protein 2-like	6	ABDE
*Cc_20225*	NAC transcription factor 29-like	6	ABCDE
*Cc_40311*	NAC domain-containing protein 62-like	8	ABCDE
*Cc_38151*	NAC domain-containing protein 2-like	6	ABCDE
*Cc_30472*	NAC transcription factor 29-like isoform X2	6	ABCDE
*Cc_30485*	NAC transcription factor 29-like	7	ABCDE
*Cc_30687*	NAC domain-containing protein 83-like	7	ABCDE
*Cc_23518*	NAC domain-containing protein 82-like	5	CDE
*Cc_42082*	NAC domain-containing protein 78	7	ABCDE
*Cc_01304*	NAC domain-containing protein 2-like	7	ABCDE
*Cc_01567*	NAC domain-containing protein 7-like	7	ABCDE
*Cc_02050*	Putative NAC domain-containing protein 94	5	CDE
*Cc_02051*	NAC domain-containing protein 41-like	7	ABCDE
*Cc_02052*	NAC domain-containing protein 41-like isoform X2	7	ABCDE
*Cc_04140*	NAC domain-containing protein 78	9	ABCDE
*Cc_48539*	NAC domain-containing protein 2, partial	5	ABD
*Cc_41044*	NAC domain-containing protein 74	6	ABD
*Cc_01440*	NAC domain-containing protein 78 (ANAC078)	7	ABDE
*Cc_26764*	NAC transcription factor ONAC010	5	BCDE
*Cc_17807*	NAC transcription factor ONAC010	2	AB
*Cc_37971*	NAC transcription factor NAM-2 (HvNAM-2)	5	ABCD
***Groundnut***			
*Ah_ann1.1I167B.2*	NAC domain-containing protein 45-like	8	ABCDE
*Ah_ann1.1Q9HM8.1*	NAC domain-containing 30	5	AB
*Ah_ann1.3GEX4P.1*	NAC domain-containing 104-like	6	ABCDE
*Ah_ann1.4435CX.1*	Protein BEARSKIN2	9	ABCDE
*Ah_ann1.4QZT53.1*	hypothetical protein Ahy_B03g067340	9	ABCDE
*Ah_ann1.5P3U81.1*	NAC domain-containing 90-like	8	ABCDE
*Ah_ann1.76LABN.1*	NAC domain-containing 86-like	8	ABCDE
*Ah_ann1.7QMU6B.1*	NAC domain-containing 83 isoform X1	7	ABCDE
*Ah_ann1.83Q9A2.1*	NAC domain-containing protein 35	5	ABE
*Ah_ann1.8AKD3R.1*	NAC transcription factor	3	AB
*Ah_ann1.A5ASCL.1*	NAC domain-containing 90-like	8	ABCDE
*Ah_ann1.AIPG34.1*	NAC transcription factor 29-like	8	ABCDE
*Ah_ann1.BX5EMB.1*	Protein CUP-SHAPED COTYLEDON 1 isoform X1	9	ABCDE
*Ah_ann1.CSHQ77.1*	NAC domain-containing protein 71	8	ABCDE
*Ah_ann1.CSZ51X.2*	NAC domain-containing 83 isoform X1	8	ABCDE
*Ah_ann1.CTTQ97.1*	Protein CUP-SHAPED COTYLEDON 1	9	ABCDE
*Ah_ann1.D5FDJH.1*	NAC transcription factor 25-like	5	ABE
*Ah_ann1.FU1JML.2*	NAC domain-containing 72-like	8	ABCDE
*Ah_ann1.G1V3KR.2*	NAC domain-containing 82-like	8	ABCDE
*Ah_ann1.GU1UJS.1*	NAC domain-containing 30	6	ABE
*Ah_ann1.JE37KP.1*	NAC domain-containing protein 35	10	ABCDE
*Ah_ann1.L9IK9Y.1*	NAC domain-containing 100	7	ABCDE
*Ah_ann1.MI72XM.2*	NAC domain-containing 82-like	8	ABCDE
*Ah_ann1.QDSH2R.1*	NAC domain-containing 104-like	6	ABCDE
*Ah_ann1.S9FEUH.1*	NAC transcription factor 29-like	8	ABCDE
*Ah_ann1.U16Y2L.1*	NAC domain-containing 86-like	8	ABCDE
*Ah_ann1.UEI6NJ.1*	NAC transcription factor	4	CDE
*Ah_ann1.V0X4SV.1*	NAC domain-containing 26-like	4	AB
*Ah_ann1.WPHD30.1*	NAC domain-containing 2	6	ABCDE
*Ah_ann1.X47CQ0.1*	NAC domain-containing 2	6	ABCDE
*Ah_ann1.YXGX3A.1*	NAC domain-containing 90-like	8	ABCDE
*Ah_ann1.YY4A03.1*	NAC domain-containing 26-like	4	AB
*Ah_ann1.ZDQ75D.1*	NAC domain-containing 90-like	8	ABCDE

### Conserved motifs and gene structure analysis of putative stress-responsive NACs

**Fig. 5:**
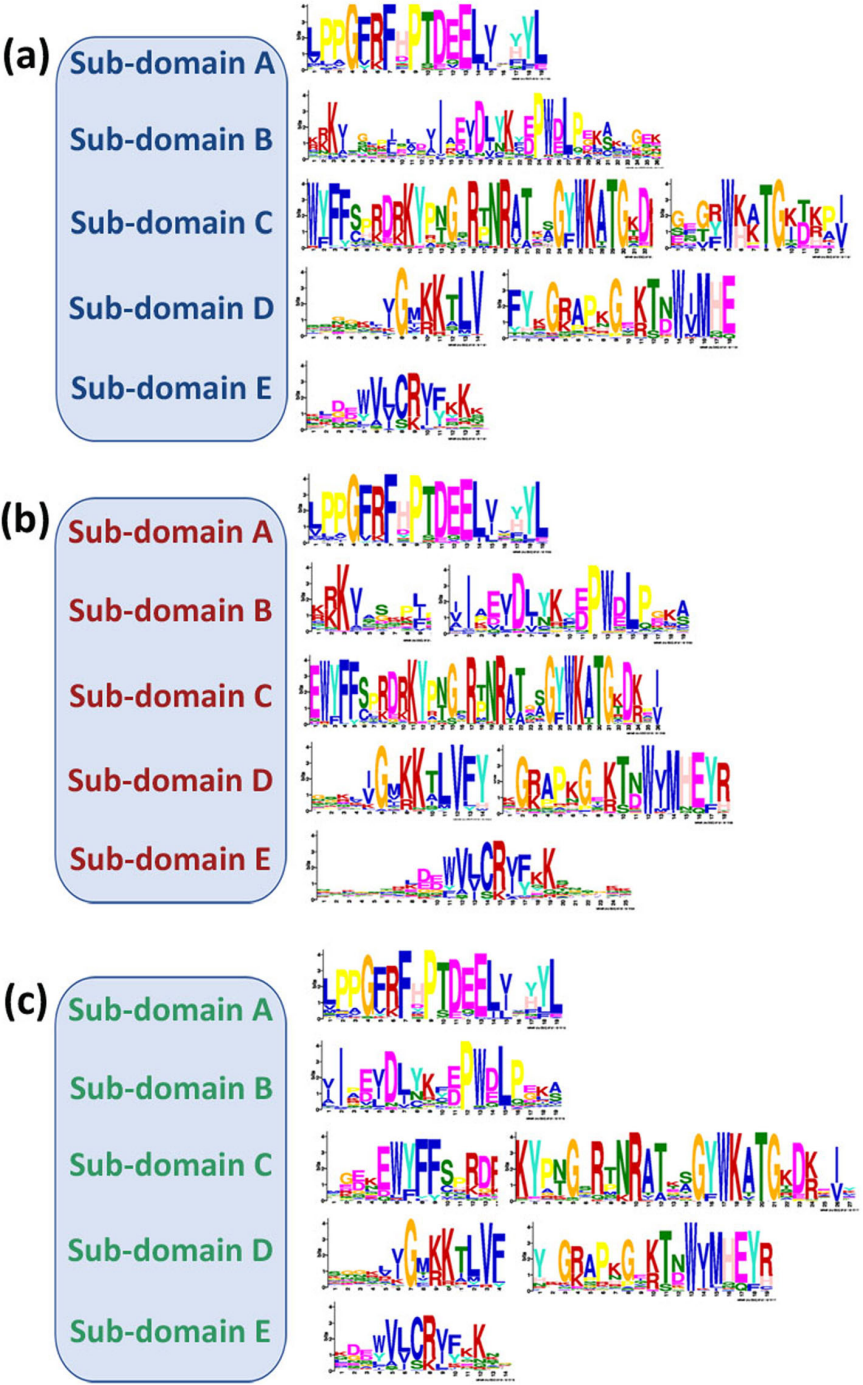
Representation of motifs of predicted stress-related NACs in three legume crops using MEME standalone version 5.0.2. The conserved motifs of NAC genes from SAT legumes (**a**) chickpea (**b**) pigeonpea (**c**) groundnut. The bit score represents the information content for each position in the sequence

All the NAC genes shared highly conserved DNA binding NAC domain consisting of five sub-domains (A-E) at the N-termini, and a variable C-terminal transcriptional regulation domain/region (TRR). Conservation of amino acid residues in NAC sub-domain (A-E) across these legumes is shown in Fig. 5a, b, c. Thus, the conserved motifs of NAC proteins were analyzed from the three SAT legumes using the MEME program (Additional file 1: Table S3). A total of 1 to 20 motifs were identified.

In addition, a detailed analysis of conserved motifs in each of the legume crop was studied (Additional file 1: Table S4). The NAC protein motifs distribution analysis revealed that 38/72 (52.8%) chickpea NAC proteins contain all five sub-domains, domains A, B, C, D and E (Additional file 1: Table S4). Interestingly, for the predicted stress-responsive genes/proteins identified, 16/22 (72.7%) contain all five NAC sub-domains A, B, C, D, E (Table 2). However, the number of motifs observed ranged from five to eight for stress-related chickpea NAC proteins. The genes *Ca_08372, Ca_04187, Ca_09673, Ca_12660, Ca_04233,* and *Ca_16946* contain the highest number of motifs (8) among the stress-related NACs, while *Ca_21186, Ca_16379, Ca_18090, Ca_05696, Ca_02365,* and *Ca_04337* have seven motifs. Five proteins, namely *Ca_05989, Ca_05227, Ca_08693, Ca_01414,* and *Ca_22941* lack one NAC sub-domain (B or C), while *Ca_06899* contains D and E NAC sub-domains only. All stress-responsive chickpea NAC proteins (22) contain sub-domain E, the most highly conserved sub-domain in chickpea NACs. Among chickpea stress-responsive NACs, only *Ca_06899* lacks NAC sub-domain A, the relatively highly conserved sub-domain. In pigeonpea, 69/96 (71.9%) NAC proteins contain all five NAC sub-domains (A-E) (Additional file 1: Table S4). Among the stress-responsive proteins, 20/31 (64.5%) NAC proteins have all five motifs (A, B, C, D, and E) (Table 2). The number of motifs identified in stress-responsive proteins ranged between two to nine in pigeonpea. Sixteen proteins (51.6%) contain seven motifs, 22.5% contain six motifs and 16% have five motifs. *Cc_01567* has the highest number of motifs observed (9) and *Cc_48539* has the least number of motifs identified (2). Six proteins lack one NAC sub-domain (A/C/E); four proteins lack two sub-domains (A and B or C and E); and only one protein, *Cc_48539*, has sub-domain A and B. The most conserved domain observed is sub-domain A and B. Only *Cc_30485* and *Cc_42082* proteins lack motifs A and B. However, motif E is the least-conserved motif (16.13%) in pigeonpea NACs. In groundnut, 99/166 (59.6%) NAC proteins have complete (A, B, C, D, and E) NAC motifs (Additional file 1: Table S4), while among the stress-responsive proteins, 25/33 (75.8%) comprise the complete NAC domain (A-E) (Table 2). In stress-responsive groundnut NACs, the number of identified motifs varied from three to ten. Fourteen out of thirty-three (42.4%) contain eight motifs, 15% has six motifs, and 12% has nine motifs. Four proteins, namely, *Ah_ann1.1Q9HM8.1, Ah_ann1.8AKD3R.1, Ah_ann1.V0X4SV.1,* and *Ah_ann1.YY4A03.1* contain A and B sub-domains only (Table 2).

**Fig. 6:**
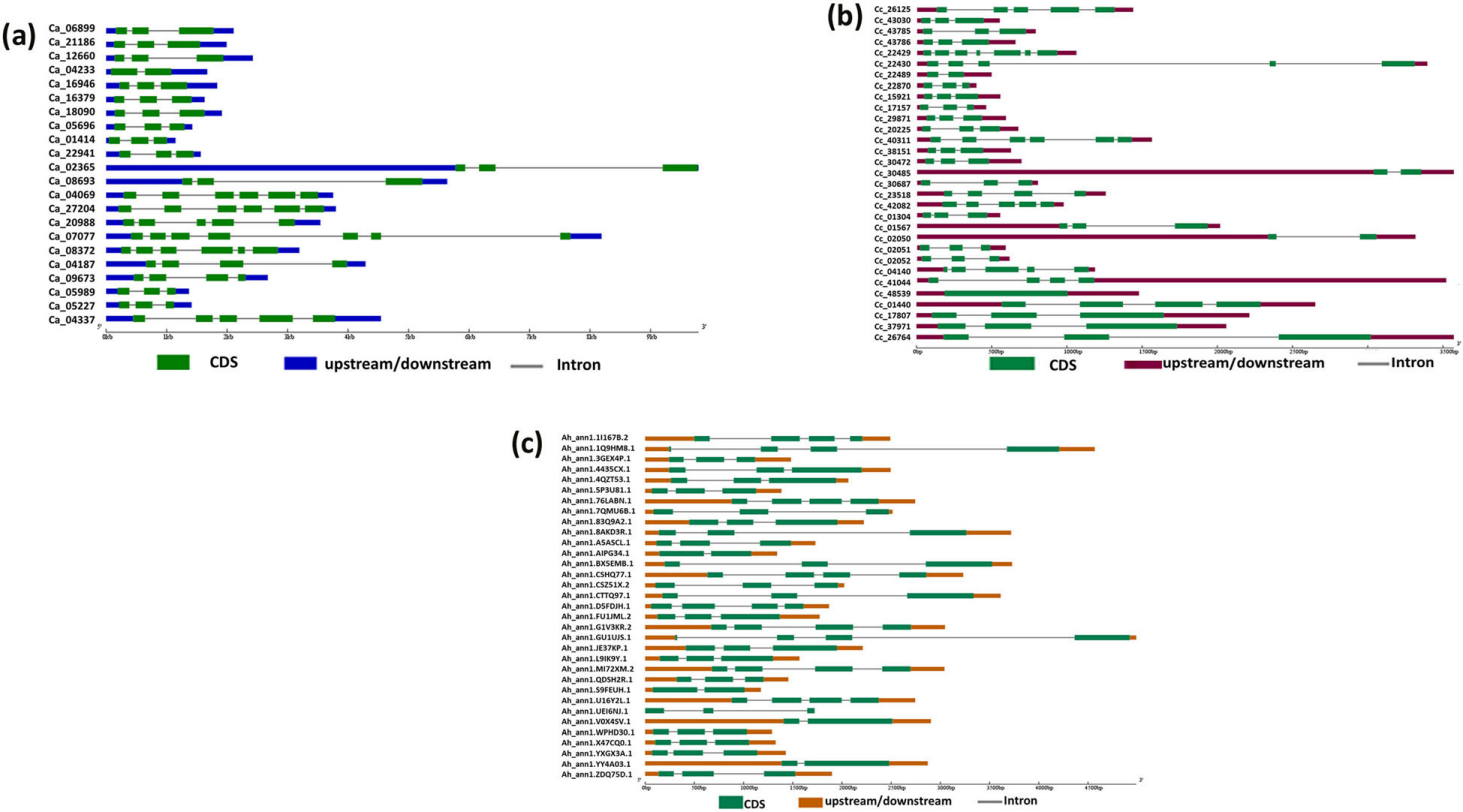
Representation of exon/intron structures of putatively predicted stress-associated NAC genes from (**a**) chickpea (**b**) pigeonpea (**c**) groundnut using GSDS 2.0 (Gene Structure Display Server). Exons and introns are represented by colored boxes and black lines, respectively. The sizes of exons and introns can be estimated using the scale below

For gene structure analysis of putative stress-responsive NAC genes in selected legume crops, the exon/intron organization of individual NAC genes was analyzed in the coding sequences of chickpea, pigeonpea and groundnut using GSDS 2.0 (Fig. 6a, b, c). Gene structure prediction revealed that the number of introns ranges from one (*Ca_04233)* to six (*Ca_07077*) in chickpea, zero (*Cc_48539*) to six (*Ca_22429*) in pigeonpea, and one to three in the groundnut NAC gene family.

### Promoter analysis of putative stress-responsive NACs

The promoter regions of NAC genes (1500-bp sequences upstream of the translational start site) were examined using the PlantCARE database to investigate transcriptional regulation and the probable functions of these putative stress-responsive NACs in chickpea, pigeonpea, and groundnut. Several *cis*-acting regulatory elements (CAREs) involved in response to drought, light, wound, developmental processes, biotic stress, tissue-specific, hormones, and other functions were discovered in the promoter regions of these NAC genes (Additional file 1: Table S5). Promoters of essential elements, such as a TATA box and a CAAT box, were predicted among all the three legumes. Of these CAREs, several regulatory elements related to tissue-specific expression, such as root-specific expression (AS1), meristem expression (CAT-box), vascular-specific expression (AC-I and AC-II motifs), and F-box (plant vegetative and reproduction growth and development; cell death and defense); and light-responsive were found widely distributed among chickpea, pigeonpea, and groundnut NAC gene promoters. Numerous CAREs involved in plant hormones, such as gibberellin-responsive elements, ABA-responsive elements (ABRE – a possible ABA-dependent regulation for abiotic stress), an ethylene-responsive element (ERE), auxin-responsive elements, MeJA-responsive elements, and salicylic acid-responsive elements (TCA) were also identified. In particular, several stress-responsive CAREs important in abiotic stress, including drought-responsive elements (MYB, MBS-MYB binding site, MYC), stress-responsive elements (STRE), dehydration-responsive elements (C repeat/DRE), and low-temperature elements (LTR) were detected. Some CAREs which function in biotic stress, including wound-responsive elements (WRE3, and WUN motif), defense- and stress-response (TC-rich repeats), and elicitor-responsiveness (W box) were also identified. In addition, promoters having zein metabolism regulation elements (O_2_-site), anaerobic induction elements (ARE element), and APETALA1 (AP1) for inducible- flowering, were observed.

The above results indicate that these NAC genes might respond to abiotic stresses and have potential roles in enhancing abiotic stress tolerance. In chickpea, thirteen NAC genes namely, *Ca_04233, Ca_16946*, *Ca_16379, Ca_18090, Ca_05696, Ca_22941*, *Ca_05696, Ca_08693, Ca_20988, Ca_07077, Ca_09673, Ca_05989* and *Ca_04337* were found to have drought-responsive elements (DRE core/MYB) (Additional file 1: Table S5). The genes, *Ca_16946, Ca_02365, Ca_07077*, *Ca_04187* and *Ca_04337* were identified as having STRE; *Ca_04233, Ca_16946, Ca_07077*, *Ca_08372, Ca_05989* and *Ca_05227* contain ABRE; *Ca_04187* and *Ca_04337* have LTR; *Ca_27204, Ca_09673* and *Ca_20988* (TC-rich repeats) have *cis*-regulatory element for defense and stress-responsiveness; *Ca_22941* and *Ca_20988* contain AE; *Ca_01414, Ca_07077*, and *Ca_04337* had elicitor responsiveness and disease resistance element (W box); *Ca_20988* contains wound-responsive element (WRE3). Furthermore, *Ca_07077* had five types of abiotic stress-responsive CAREs viz., DRE core, STRE, MYB, ABRE, and W box. *Ca_04337* contains W box, STRE, MYB, LTR, and MYC types of *cis*-elements. In general, almost all the putative *Ca_NACs* contain at least two or more different types of stress-responsive CAREs. Some tissue-specific CAREs, such as AS1 were identified in *Ca_16946, Ca_22941, Ca_07077, Ca_08372*, *Ca_04187, Ca_09673,* and *Ca_05227* NAC genes; AC-I (vascular-specific expression) was detected in *Ca_07077*; AP1 (flowering inducible) was found in *Ca_08372*.

Among pigeonpea NAC genes, nine genes had MYB binding site, eight had ABRE, nine had ARE, 10 had STRE, five had W box, three had WUN motif/WRE3, and two had LTR (Additional file 1: Table S5). For instance, *Cc_26125, Cc_41044* and *Cc_01567* genes had up to five different types of abiotic stress-related CAREs. Furthermore, STRE, ARE, MBS, W box, and LTR were found in *Cc_26125*. Similarly, *Cc_41044* contains DRE core, ARE, STRE, MBS/MYB and ABRE; while W box, ARE, MYB, MBS, and MYC CAREs were predicted in *Cc_01567*. In addition, some of these genes have three types of stress-associated CAREs. *Cc_04140* had W box, STRE, and ABRE; *Cc_22489* and *Cc_15921* had ARE, LTR, and ABRE abiotic stress-associated motifs. Moreover, 12 of the 31 genes were predicted to have two types of stress-associated *cis*-elements. *Cc_22870* and *Cc_38151* had WRE3 and ABRE; *Cc_22430* possessed STRE and W box; *Cc_17157* and *Cc_20225* contained STRE and ARE; *Cc_29871* and *Cc_48539* had ABRE and STRE; and contain WRE3 and ABRE; *Cc_23518* contains DRE core and MYB; *Cc_42082* and *Cc_37971* contained ARE and MYB recognition site; *Cc_04140* had WUN-motif and MYB/MBS/MYC; and *Cc_26764* had STRE and MBS/MYB abiotic stress-associated CAREs. With regard to tissue-specific expression, AS1 element (root-specific expression) was noted in *Cc_22870*, *Cc_29871*, *Cc_01567*, *Cc_04140* and *Cc_48539*; CAT-box (meristem-specific expression) was reported in *Cc_23518*, *Cc_04140, Cc_48539*, *Cc_26125*, and *Cc_29871* genes; and AC-II, AC-I (vascular expression) were reported in *Cc_04140*.

In groundnut, 17 NAC genes were identified as having drought-responsive elements (MYB-like sequence, MYB/MYC/DRE core) (Additional file 1: Table S5). Five genes were reported to have ABRE3a/4/ABRE (*Ah_ann1.3GEX4P.1, Ah_ann1.FU1JML.2, Ah_ann1.QDSH2R.1, Ah_ann1.WPHD30.1,* and *Ah_ann1.X47CQ0.1*); LTR (*Ah_ann1.1I167B.2, Ah_ann1.A5ASCL.1, Ah_ann1.CSHQ77.1*, *Ah_ann1.U16Y2L.1*, and *Ah_ann1.ZDQ75D.1*), and WUN-motif (*Ah_ann1.1I167B.2*, *Ah_ann1.76LABN.1, Ah_ann1.CSHQ77.1, Ah_ann1.QDSH2R.1,* and *Ah_ann1.U16Y2L.1*). Four genes, *Ah_ann1.1I167B.2, Ah_ann1.3GEX4P.1, Ah_ann1.CSHQ77.1*, and *Ah_ann1.QDSH2R.1* had W box element. TC-rich repeats were reported in *Ah_ann1.U16Y2L.1* and *Ah_ann1.1I167B.2*. Seven different types of abiotic stress-related motifs, MYB/MBS/MYC, STRE, LTR, DRE core, TC-rich repeats, W box, and ARE could be seen in *Ah_ann1.1I167B.2*. W box, MYB-like sequence, WUN-motif, STRE, ARE, LTR and MYB were observed in *Ah_ann1.CSHQ77.1*. Five types of motifs, MYB-like sequence, WUN-motif, STRE, TC-rich repeats, and LTR are found in *Ah_ann1.U16Y2L.1*. Four types of abiotic stress CAREs ABRE3a/ABRE4/ABRE, MYB, W box, and WUN-motif were seen in *Ah_ann1.QDSH2R.1*. Moreover, *Ah_ann1.3GEX4P.1* and *Ah_ann1.4435CX.1* had motifs for drought-responsiveness (W box, ABRE3a/ABRE4/ABRE, MYB/ DRE core, and WRE3). In terms of tissue specificity, four proteins had AS1 and AP1.

### Protein-protein interaction network analysis

The predicted protein-protein interaction map displayed interactions among themselves and with several other proteins. NAC protein sequences of the three legumes were searched against *Arabidopsis* proteins for the best possible match and the corresponding proteins were further used for network analysis (Additional file 1: Tables S6, S7). Several strong interaction/s could be noticed, for e.g., *ATAF1* (*Cc_01304, Cc_29871, Cc_48539, Ca_12660* and *Ca_16946*), which increases in response to wounding and abscisic acid with *NAC102* (*Cc_15921*) that functions in response to hypoxia in germinating seedlings. Likewise, *NAC062* (*Cc_42082*) which is induced in response to cold stress, showed strong association with *CZF1* (salt stress-response), *BZIP60* (ER stress-response), *SZF1* (salt stress-response) and *NTL* (protein transporter activity) (Additional file 2: Fig. S4). Another vital interaction observed was *NAC007* (*Cc_01567, Ca_02365,* and *Ah_ann1.V0X4SV.1*), a transcriptional activator that binds to the secondary wall NAC binding element with *VND7* (*Ah_ann1.1Q9HM8.1* and *Ah_ann1.GU1UJS.1*) (xylem formation in roots and shoots), *MYB46* (regulation of secondary wall biosynthesis in fibers and vessels), and *MYB83* (molecular switch in the *NAC012/SND1*-mediated transcriptional network regulating secondary wall biosynthesis). Similarly, *XND1* (*Cc_17157, Cc_22870, Ca_05227, Ah_ann1.3GEX4P.1,* and *Ah_ann1.QDSH2R.1*), which regulates secondary cell wall fiber synthesis and programmed cell death, displayed strong relationship with *MYB46* and *MYB83*, while *NAP* (*Cc_20225, Cc_30472, Cc_30485, Ca_16379, Ca_18090, Ah_ann1.AIPG34.1,* and *Ah_ann1.S9FEUH.1*) that has a role in controlling dehydration in senescing leaves showed interactivity with *NAC6* (promotes lateral root development; triggers the expression of senescence-associated genes). Likewise, *NAC014* (*Ca_20988*) (transcriptional activator) interacts with *AT1G49560* (phosphate signaling in roots) (Additional file 2: Fig. S4).

### Expression pattern of putative stress-responsive NACs across different developmental tissues in chickpea, pigeonpea and groundnut

**Fig. 7:**
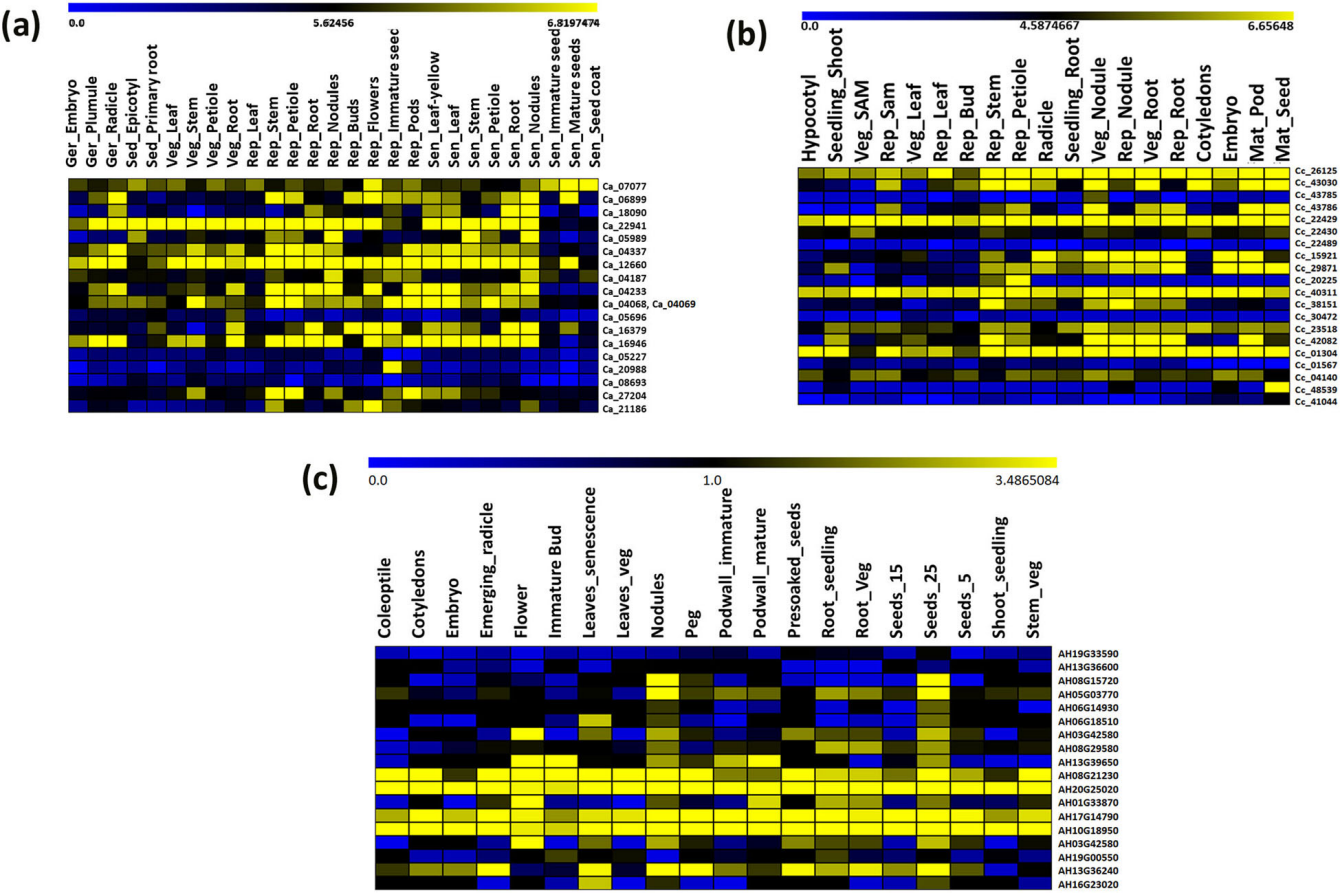
Representation of heatmaps viewed in MeV tool version 4.9.0 for expression patterns of identified stress-responsive NAC genes of the three legume crops. **a** Heatmap representation for expression of identified stress-responsive *Ca_NAC* genes across different tissues from germinating, seedling, vegetative, reproductive and senescence stages in chickpea. The expression data generated by RNA sequencing of plumule, radicle, shoot, leaf, bud, stem, nodule, root, etc., tissues at various stages were obtained from *Cicer arietinum* gene expression atlas (CaGEA) [35]. Yellow and blue color gradients indicate an increase or decrease, respectively, in transcript abundance represented in log_2_ values. Ger-germinating; Sed-seedling; Veg-vegetative; Rep-reproductive; Sen-senescence. **b** Heatmap representation for expression of putative stress-responsive *Cc_NAC* genes in various tissues of pigeonpea. The expression data generated by Illumina sequencing of RNA-seq libraries prepared from shoot, leaf, stem, root, bud, nodule, embryo, seed, pod, etc., tissues across different stages were obtained from *Cajanus cajan* gene expression atlas (CcGEA) [36]. Yellow and blue color gradients indicate an increase or decrease, respectively, in transcript abundance represented in log_2_ values. Veg-vegetative; Rep-reproductive; SAM-shoot apical meristem; Mat-mature. **c** Heatmap showing expression of predicted stress-responsive *Ah_NAC* genes in various tissues at different stages (germinal, seedling, vegetative reproductive, and senescence) of groundnut. The expression data generated by Illumina sequencing of RNA-seq libraries prepared from cotyledon, embryo, shoot, root, bud, nodule, embryo, seed, pod wall, etc., tissues at different stages were obtained from *Arachis hypogea* gene expression atlas (AhGEA) [37]. Yellow and blue color gradients indicate an increase or decrease, respectively, in transcript abundance represented in log_2_ values. Veg-vegetative; Seeds_5-seeds after 5 days of planting; Seeds_25- seeds after 25 days of planting

Expression profiles for putatively predicted stress-responsive NAC genes possessing varied transcript abundance in various tissues at different growth stages of the plant (germination, seedling, vegetative, reproductive and senescence) is represented in the form of a heat map generated from comprehensive Gene Expression Atlases viz., CaGEA, CcGEA, and AhGEA for chickpea [35], pigeonpea [36] and groundnut [37], respectively (Fig. 7a, b, c). Nineteen of 22 chickpea NACs, 20 of 31 pigeonpea NACs, and 18 of 33 groundnut NACs were found expressed in their respective gene expression atlases. Majority of the putative stress-responsive NAC genes are among those with high transcript abundance observed in the tissues, in almost all legume crops studied. Genes *Ca_07077, Ca_06899*, *Ca_22941*, *Ca_04337*, *Ca_12660*, *Ca_04068*, *Ca_04069*, *Ca_16946*, *Ca_16379* and *Ca_04233* had high transcript abundance examined across tissues in CaGEA. A few of the NAC genes were tissue-specific viz., *Ca_05696* (vegetative root), *Ca_20988* (immature seeds and pods) and *Ca_08693* (seedling epicotyl, senescence stem and root), while most of them were found to be ubiquitously expressed (*Ca_07077, Ca_22941, Ca_04337, Ca_12660, Ca_04068, Ca_04069,* and *Ca_16946*) across all the tissues (Fig. 7a). In pigeonpea, *Cc_26125*, *Cc_22429*, *Cc_22430, Cc_15921, Cc_29871, Cc_40311, Cc_38151, Cc_23518, Cc_01304, Cc_42082* and *Cc_04140* NAC genes were found to have high transcript accumulation and were expressed ubiquitously across various tissues studied (Fig. 7b). Genes, such as *Cc_43578* (vegetative nodule)*, Cc_22489, Cc_20225* (reproductive stem and petiole)*, Cc_30472* and *Cc_48539* (mature seeds) were found to be tissue-specific. In case of groundnut (Fig. 7c), all the NAC genes (out of 18) were found to have high transcript levels at least in some of the tissues, except for AH19G33590 (*Ah_ann1.1Q9HM8.1*)*,* AH13G36600 (*Ah_ann1.QDSH2R.1* and *Ah_ann1.3GEX4P.1*)*,* and AH19G33590 (*Ah_ann1.GU1UJS.1*) genes. Genes, such as AH20G25020 (*Ah_ann1.G1V3KR.2*), AH10G18950 (*Ah_ann1.MI72XM.2*), AH17G14790 (*Ah_ann1.MFVS6B.1*), and AH08G21230 (*Ah_ann1.FU1JML.2*) are among those which appeared to be ubiquitously expressed across all the tissues. Genes, namely AH08G15720 (*Ah_ann1.5P3U81.1* and *Ah_ann1.YXGX3A.1*) (seeds_25 and nodules), AH06G18510 (*Ah_ann1.A5ASCL.1*) (seeds_25, nodules, and senescence leaves), AH06G14930 (*Ah_ann1.8AKD3R.1*) (seeds_25 and nodules), AH16G23020 (*Ah_ann1.ZDQ75D.1*) (seeds_25 and nodules), and AH19G00550 (*Ah_ann1.YY4A03.1* and *Ah_ann1.V0X4SV.1*) (vegetative leaves, immature bud, and root seedlings) were found to be tissue-specific. However, genes such as AH13G39650 (*Ah_ann1.D5FDJH.1*), AH01G33870 (*Ah_ann1.L9IK9Y.1*), AH05G03770 (*Ah_ann1.76LABN.*1 and *Ah_ann1.U16Y2L.1*), and AH08G29580 (*Ah_ann1.7QMU6B.1*) were found expressed in most of the tissues, including immature bud, flower, seeds_25, nodules, immature and mature pod wall, etc. Interestingly, all the genes except AH19G33590 (*Ah_ann1.1Q9HM8.1* and *Ah_ann1.GU1UJS.1*), AH13G36600 (*Ah_ann1.QDSH2R.1* and *Ah_ann1.3GEX4P.1*), AH19G00550 (*Ah_ann1.YY4A03.1* and *Ah_ann1.V0X4SV.1*) were highly expressed at stage seeds_25 and nodules in the groundnut gene expression atlas. Details of corresponding transcript ids for groundnut NAC genes are provided in the Additional file 1: Table S8.

In summary, based upon the expression of these genes in root tissues – whether primary, vegetative or reproductive (radicle and nodules also) – fifteen genes in each legume crop were selected for validation of expression patterns under control and drought stress condition of contrasting drought-responsive genotypes. For instance, the genes- *Ca_07077*, *Ca_22941*, *Ca_05989*, *Ca_04337*, *Ca_12660*, *Ca_04187*, *Ca_04069*, *Ca_04233*, *Ca_16379*, *Ca_16946*, *Ca_27204*, *Ca_06899*, *Ca_18090*, *Ca_21186* and *Ca_05227* were identified in chickpea across tissues, such as radicle (germination stage), primary root (seedling), root (vegetative, reproductive and senescence) and nodule (senescence). Similarly, expression in tissues like radicle, primary root, vegetative and reproductive root tissues were analyzed for pigeonpea, and the genes identified – *Cc_26125*, *Cc_43030, Cc_43785*, *Cc_43786*, *Cc_22429*, *Cc_22430*, *Cc_22489*, *Cc_15921*, *Cc_29871*, *Cc_40311*, *Cc_38151, Cc_23518*, *Cc_42082*, *Cc_01304*, and *Cc_04140* – for validation of their expression profiles under drought stress condition. Further, radicle, primary root, vegetative root and nodules were targeted, and the following genes: *Ah_ann1.V0X4SV.1*, *Ah_ann1.7QMU6B.1*, *Ah_ann1.76LABN.1*, *Ah_ann1.L9IK9Y.1*, *Ah_ann1.D5FDJH.1*, *Ah_ann1.G1V3KR.2*, *Ah_ann1.MI72XM.2*, *Ah_ann1.X47CQ0.1*, *Ah_ann1.AIPG34.1*, *Ah_ann1.WPHD30.1*, *Ah_ann1.FU1JML.2*, *Ah_ann1.8AKD3R.1*, *Ah_ann1.A5ASCL.1*, and *Ah_ann1.5P3U81.1*, were selected for validation in groundnut.

### Validation of predicted stress-responsive NAC genes under induced drought treatment

**Fig. 8:**
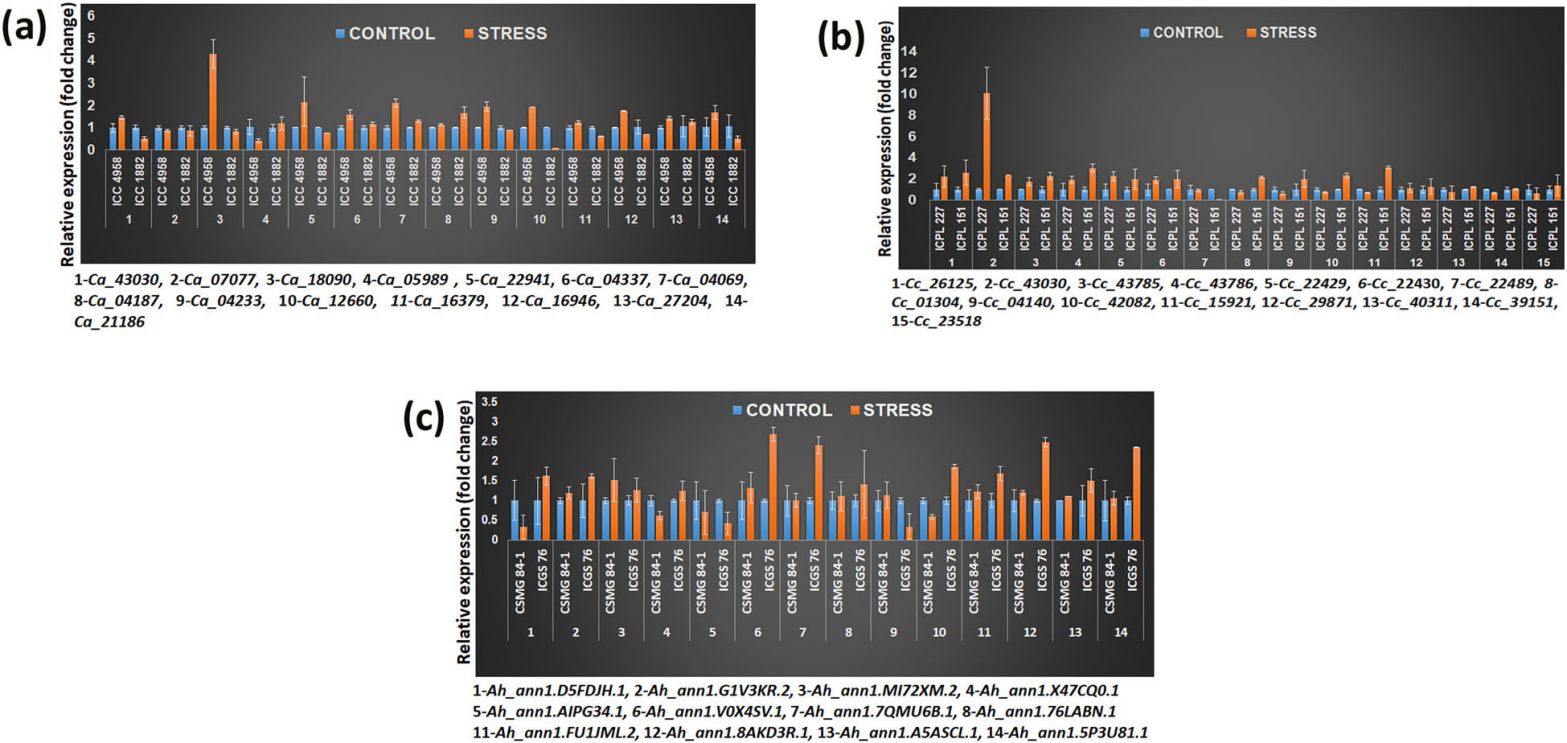
Validation of expression profiles of selected NAC genes in contrasting drought-responsive genotypes of the three legume crops. **a** Expression of selected *Ca_NAC* genes in chickpea root tissues under drought stress treatment. Expression data were obtained by qRT-PCR of drought-stressed and well-watered root samples of 30-day-old chickpea plants. Root tissues were collected after six days of drought induction. Mean relative expression levels were normalized to a value of 1 in control root samples. Fourteen of fifteen selected genes (except *Ca_05227*) were examined. Error bars = SE values of two biological replicates and three technical replicates. Significant differences were determined by Student’s t-test at *P* ≤ 0.05. **b** Expression of selected *Cc_NAC* genes in pigeonpea root tissues under drought stress treatment. Expression data were obtained by qRT-PCR of drought-stressed and well-watered root samples of 30-day-old pigeonpea plants. Root tissues were collected after six days of drought induction. Mean relative expression levels were normalized to a value of 1 in control root samples. Error bars = SE values of two biological replicates and three technical replicates. Significant differences were determined by Student’s t-test at P ≤ 0.05. **(c)** Expression of selected *Ah_NAC* genes in groundnut root tissues under drought stress treatment. Expression data were obtained by qRT-PCR of drought-stressed and well-watered root samples of 30-day-old groundnut plants. Root tissues were collected after six days of drought induction. Fourteen selected genes were examined. Mean relative expression levels were normalized to a value of 1 in control root samples. Error bars = SE values of two biological replicates and three technical replicates. Significant differences were determined by Student’s t-test at P ≤ 0.05

To assess the potential and response of these stress-responsive NACs under drought stress (PEG 8000 exposure), two contrasting genotypes for each crop were selected and analyzed the expression patterns of these genes in root tissues using quantitative real time PCR (qRT-PCR). Chickpea genotypes - ICC 4958 (tolerant) and ICC 1882 (sensitive); pigeonpea genotypes - ICPL 227 (tolerant) and ICPL 151 (sensitive); and groundnut genotypes - CSMG 84–1 (tolerant) and ICGS 76 (sensitive) were selected for validation of expression profiles of identified candidate NACs. Details of the selected primer pairs are provided in Additional file 1: Table S9. These results indicated that majority of these genes (12 of 15) showed up-regulation with a maximum fold change of 4.3 (*Ca_18090*) in tolerant genotype, ICC 4958, while only two genes, *Ca_07077* (0.87 folds) and *Ca_05989* (0.4 folds) showed down-regulation with respect to their controls, under drought stress in chickpea. However, a few genes viz., *Ca_05989* (1.19 folds), *Ca_04337* (1.16 folds), *Ca_04069* (1.29 folds), *Ca_04187* (1.65 folds) and *Ca_27204* (1.27 folds) were found slightly up-regulated in susceptible genotype (ICC 1882), though the expression was not higher than the tolerant genotype (ICC 4958) except for *Ca_04187* gene which showed higher expression than the tolerant one (Fig. 8a). Further, for pigeonpea, all the selected 15 NAC genes examined were found to be up-regulated with a maximum of 3.1 folds (*Cc_15921*) in the case of ICPL 151, except *Cc_22489* gene which was down-regulated in both the genotypes under drought stress (Fig. 8b). However, the genes *Cc_26125, Cc_43030*, *Cc_43785*, *Cc_43786*, *Cc_22429*, and *Cc_22430* were found up-regulated for ICPL 227 (more drought-tolerant) genotype with a maximum of 10 folds up-regulation (*Cc_43030*). In the case of groundnut, the relative fold change expression was up-regulated in 10 genes for CSMG 84–1 genotype, while 12 genes displayed up-regulation in ICGS 76 genotype with respect to their control (Fig. 8c). Nine genes viz., *Ah_ann1.G1V3KR.2*, *Ah_ann1.MI72XM.2*, *Ah_ann1.V0X4SV.1*, *Ah_ann1.7QMU6B.1*, *Ah_ann1.76LABN.1*, *Ah_ann1.FU1JML.2*, *Ah_ann1.8AKD3R.1*, *Ah_ann1.A5ASCL.1*, and *Ah_ann1.5P3U81.1* were found up-regulated in both the genotypes in response to drought stress.

## Discussion

Chickpea, pigeonpea, and groundnut are important food legumes, particularly in SAT regions. The seeds of these legumes are an essential food source, while the crop plants also contribute to the fertility of the soil. Furthermore, genome sequences have been available for several food legumes, including pigeonpea [7], chickpea [5], mung bean [38], common bean [39], adzuki bean [40], and groundnut [8, 9]. The genome sequence of chickpea was from CDC Frontier, a Canadian ‘*kabuli’* variety [5] and ‘*desi’* ICC 4958 cultivar [41], pigeonpea genome was from the genotype ICPL 87119, popularly known as Asha [7], and groundnut genome from *A. hypogaea* cv Tifrunner (CV-93, PI 644011), a runner-type groundnut habituated to the southeast of the United States of America [8]. The progress in genome sequencing has provided valuable genomic resources for comparative genomic analyses in these sequenced food legume crops [42]. Being one of the largest among plant-specific TFs, the NAC protein family has a role in plant development, abiotic stress and defense responses. In many plant species NAC proteins have been functionally characterized, including those of *Arabidopsis thaliana*, *Oryza sativa*, *Zea mays*, *Triticum aestivum, Glycine max*, *Populus trichocarpa* and other plants [15, 43–45]. However, the functions for majority of the NAC genes in legume crops remain unknown. In the present study, genome-wide identification of NAC domain TFs has been performed to identify and characterize drought-responsive NAC proteins encoded in chickpea, pigeonpea, and groundnut genome.

Similar studies were conducted in other plant species, for example, 163 NAC genes in *Populus* [44], 140 in *Oryza* [43], 105 in *Arabidopsis* [15], and 101 in *Glycine max* [45] were analyzed. In this study, a total of 72, 96 and 166 non-redundant NAC genes were analyzed from chickpea, pigeonpea and groundnut, respectively. The number of NAC genes identified in chickpea and pigeonpea is low when compared to those assessed for other plant species such as, Arabidopsis, rice, maize and soybean. Typically, chickpea (~ 738.09 Mb) and pigeonpea genomes (~ 833 Mb) are much larger than that of Arabidopsis (125 Mb), and rice (480 Mb), indicating that the number of NAC genes is not directly correlated with genome size.

The identified NAC genes varied greatly in protein length, from 56 AA to 740 AA residues in these three legumes. Average length of the identified NAC proteins was 321.3 AA in chickpea, 330.9 AA in pigeonpea, 321.2 AA in groundnut. As mentioned above, NAC domain is approximately 160 amino acid in length, despite these few small NAC TFs/genes were found such as, *Ca_15515* (106 AA), *Cc_48539* (56 AA), *Ah_ann1.8AKD3R.1* (62 AA), those still encodes NAC domain in the three legumes. Furthermore, *Ca_15515* consists of only A sub-domain, while *Ah_ann1.8AKD3R.1* and *Cc_48539* genes contain A and B sub-domains. Similarly, NAC protein with 106 amino acid residues has been reported by in case of *CaNAC2* gene in *Capsicum* [46]. Mohanta et al. [47] reported the smallest NAC TF of only 25 amino acids in *Fragaria* × *ananassa* (strawberry) that codes NAC domain.

Subcellular localization prediction revealed 62/96 of identified NAC genes in pigeonpea, 53/72 genes in chickpea, and 96/166 genes in groundnut were potentially located in the nucleus. Rest of the NAC genes were localized extracellularly or secreted in these legumes. Transcription factors need to be localized to nucleus to execute their function, either independently or by interacting with other partners. For instance, *ATAF1* is localized to nucleus [48], whereas *ONAC020* and *ONAC023* completely gets localize to nucleus after interacting with *ONAC26* [28]. Similarly, *NTL4* is targeted to the nucleus only upon heat stress after processing [49]. There are numerous reports which shows the localization of NAC genes in different organelles other than nucleus, as in case of *MaNAC6* which gets localized to the cell membrane, cytoplasm and nucleus [50], and *ONAC023* is localized to the cytoplasm [28]. Sometimes, TFs gets localized to nucleus after splicing of membrane bound TFs or upon proteolytic cleavage [51, 52].

Ten major groups for legumes – chickpea (Fig. 3a), pigeonpea (Fig. 3b) – and 12 groups for groundnut NACs have been identified (Fig. 3c) based on phylogenetic tree analysis. Earlier studies reported 12 groups in chickpea using 71 NAC proteins [53] and seven major groups in pigeonpea using 88 NAC proteins [54]. Similarly, eight major groups were reported in common bean NACs [55], 15 groups in tartary buckwheat NACs, 16 groups in cassava NACs, and 14 in pepper NACs, 12 NAC groups in broomcorn millet [56]. Furthermore, a comprehensive study of 11 different species with a total of 1232 NAC proteins classified them into eight subfamilies [57]. By analyzing gene structures of the putative stress-responsive NAC proteins, it was observed that predicted NAC genes contained one to six introns in chickpea, zero to six in pigeonpea, and one to three in groundnut. Similarly, introns of common bean NAC genes ranged from one to five [55] and *Glycine max* NAC genes from one to seven [58]. However, in rice, poplar and cotton, NAC genes introns vary from 0 to 16 [59], 0–8 [44] and 0–9 [60], respectively. Interestingly, the majority of the chickpea (13), pigeonpea (15), and groundnut (18), stress-responsive NACs have two introns. In general, highly similar NAC gene structures were clustered in the same group of their respective phylogenetic trees. However, the distribution of the conserved motif in NAC genes of SAT legumes was similar to that of other species, including common bean, rice, soybean, and *Arabidopsis*. The sub-domain A has a role in dimer formation, while sub-domain D contains the nuclear localization signal. The most conserved sub-domains are C and D and positively charged, whereas the relatively divergent sub-domains are B and E which may be contributing to functional diversity along with the C-terminal domains of NAC proteins [14]. Among the stress-responsive NACs, the highly conserved sub-domain observed is E, whereas sub-domain A is relatively highly conserved in chickpea. In pigeonpea sub-domain E is least conserved, while sub-domain A and B are most conserved. Interestingly, 73, 64.5, and 75.8% of stress-responsive NACs had all five sub-domains in chickpea, pigeonpea, and groundnut. Despite that, the diversity of gene structures and conserved motifs also implies that these legume NAC proteins are functionally diverged, having roles in meristem development, root development, flowering-inducible, embryo development, vascular-specific expression, hormone signaling, abiotic stresses and defense responses.

To identify putative abiotic stress-responsive NAC genes/proteins in the selected legume crops, we proceeded with the fact that similar protein sequences have similar functions [61]. Thus, the predicted abiotic stress-responsive NAC genes were identified and the functions were analyzed based on the phylogenetic analysis. For this, a total of 107, 139, and 209 NAC protein sequences were used for chickpea, pigeonpea, and groundnut, respectively, including 43 well-known stress-responsive NACs from model and crop plants (*Arabidopsis thaliana*, *Oryza sativa*, *Medicago truncatula* and *Glycine max*). As most of the *ANACs* [62–64], *ONACs* [43, 65, 66], *MtNACs* [67], and *GmNACs* [58] included in the phylogenetic analysis have known functions in stress responses, 22, 31, and 33 abiotic stress-responsive NAC genes in chickpea, pigeonpea, and groundnut, respectively, were identified in the present study. There could be more stress-responsive NACs, dispersed on different branches if more stress-responsive NAC proteins from model plants and crops (*ANAC*, *ONAC*, *MtNAC*, and *GmNAC*) were used – as demonstrated in soybean [61]. Considering this tree-based approach, the possible role of *Ca_16946* and *Cc_38151* in cold- and drought stress response has been predicted as they are clustered into one subgroup with *Medtr8g094580.1* [67]. Similarly, *Gm_NAC066*, *Ca_12660,* and *Cc_01304* clustered into the same subgroup; *Gm_NAC065* and *Cc_29871* in one group; *Cc_48539* and *LOC_Os03g60080.1* in one subclass indicate that these genes may be involved in drought stress response [58]. Some genes may have a role in response to cold stress, like *Medtr8g059170.1* and *Ca_21186*. Genes *Ca_18090* and *Medtr5g041940.1* contribute to many stresses such as cold, drought, salicylic acid and ABA induced abiotic stress response. Genes, such as *Ca_05696*, *Cc_30687*, and *Medtr8g099750.1*; and *Cc_04140*, *Ca_04187*, *Ah_ann1.G1V3KR.2*, *Ah_ann1.MI72XM.2*, and *Medtr3g096140.2* are closely related and have been supposed to be involved in salt and drought stress [67]. Furthermore, *LOC_Os01g15640.1* and *Ah_ann1.UEI6NJ.1* have been reported to be involved in multiple abiotic stresses, such as drought, cold, salinity, and heat [43, 65]. Detailed characterization of the gene composition in legumes using comparative genomics is feasible for deriving functional insights of key candidate genes.

*Cis*-acting regulatory elements (CAREs) are among the most critical gene structures, which determine the transcriptional initiation and consist of short conserved motifs (5 to 20 nucleotides) found in the upstream of the transcriptional start codon [68]. In this study, 13, 9, and 17 drought stress-responsive CAREs were identified in chickpea, pigeonpea, and groundnut, respectively. Another important CAREs detected is Abscisic acid Response Element (ABRE) for abiotic stress regulation which was 06 in chickpea, 08 in pigeonpea, and 05 in groundnut. Also 5, 10, and 10 stress-responsive elements (STRE) in chickpea, pigeonpea and groundnut, respectively, were observed. Besides these, several other promoter elements were also identified which have a role in various plant development and stress response. CAREs are necessary for stress-responsive transcriptional regulation [69]. The existence of different *cis*-regulatory elements indicates the transcription of several stress-responsive genes via a variety of TFs. Moreover, the significance of the association between CAREs has already been documented for stress-responsive transcription [70]. Hence, the availability of diverse stress-associated elements in the putative stress-responsive NACs deduced from phylogeny might have a role in conferring drought stress tolerance in these legume crops. In several reports, various *cis*-motifs as DNA-binding sites for the NAC TFs have been identified, which include NACRS (NAC-recognition sequence for drought response) [62], IDE2 motif (iron deficiency-responsive) [71], SNBE (secondary wall NAC binding element) [21], and calmodulin-binding (CBNAC) [72]. As NAC TFs are multiple functional proteins, they can use their DNA binding NAC domains for mediating protein-protein interactions as well [73]. This study showed strong protein-protein interactions between *Cc_42082*, *CZF1*, *SZF1* (salt-stress response), *BZIP60* (ER-stress response), and *NTL* (protein transporter activity); *Cc_01567, Ca_02365, Ah_ann1.V0X4SV.1*, *VND7*, *MYB46*, and *MYB83* (regulation of secondary wall biosynthesis).

Furthermore, candidate NAC genes were identified, especially the drought-related NACs. Transcript abundance analysis for particular NAC genes (15 from each legume) was performed upon drought exposure in root tissues. For expression analysis, genes were selected based on their expression patterns in root tissues from different developmental stages of the plant available from gene expression atlases of chickpea [35], pigeonpea [36] and groundnut [37]. Real-time qRT PCR-based gene expression was also analyzed among drought-tolerant and sensitive genotypes. In chickpea, drought-tolerant genotype (ICC 4958) exhibited higher transcript levels when compared to the sensitive genotype (ICC 1882). However, *Ca_04337*, *Ca_04187, Ca_04069 and Ca_27204* were found up-regulated irrespective of the drought sensitivity of the genotype, though the expression levels were observed as being lower (except *Ca_04187*) than the tolerant genotype under stressed conditions. In pigeonpea, seven genes (*Cc_29871*, *Cc_26125*, *Cc_43030*, *Cc_43785*, *Cc_43786*, *Cc_22429*, and *Cc_22430*) were found to be induced in both the genotypes ICPL 227 and ICPL 151, under drought stress. Interestingly, a total of 13 genes (out of the 15 examined) were found to be up-regulated in ICPL 151, less drought-tolerant genotype against drought stress, and have greater expression levels than ICPL 227 for majority of the genes – confirming that pigeonpea is a relatively drought-tolerant crop. Similarly, in the case of groundnut, nine genes were up-regulated in both tolerant (CSMG 84–1) and susceptible (ICGS 76) genotypes. Comparing the expression of these genes (homologs) in crops, such as *Arabidopsis* and *Oryza* revealed their strong induction in several abiotic stresses including salinity, drought, cold, heat, and were mostly up-regulated during high drought, salinity and heat stresses (Additional file 1: Table S4). Moreover, experimental evidences showed that they are expressed in roots, rosette leaves, cauline leaves, shoot apex, stems and flowers [62, 74]. However, *Medtr8g094580.*1 showed down-regulation in response to drought stress in *Medicago* [67]. Similarly, lesser transcript level of *Ca_16946* (*Medtr8g094580*) was observed in sensitive cultivar (ICC 1882) in drought response. Interestingly, *Ca_04337, Ca_04069, Ca_27204, Cc_26125, Cc_22429, Cc_42082,* and *Cc_40311* are membrane-bound NAC proteins. These proteins are known to be primarily localized in plasma membrane/endoplasmic reticulum membrane in dormant form and processed into a transcriptionally active and nuclear form after proteolytic cleavage via regulated intramembrane proteolysis, upon specific stress [75, 76].

Drought stress has altered the expression of many NAC genes in these legumes. Thus, based on drought-induced expression of 15 genes examined in each of the three legumes, the possible role of 10 (*Ca_06899, Ca_18090, Ca_22941, Ca_04337, Ca_04069, Ca_04233, Ca_12660, Ca_16379, Ca_16946,* and *Ca_21186*), 06 (*Cc_26125*, *Cc_43030*, *Cc_43785*, *Cc_43786*, *Cc_22429*, and *Cc_22430*), and 05 (*Ah_ann1.G1V3KR.2*, *Ah_ann1.MI72XM.2*, *Ah_ann1.V0X4SV.1*, *Ah_ann1.FU1JML.2*, and *Ah_ann1.8AKD3R.1*) potential NAC genes in drought stress response of chickpea, pigeonpea, and groundnut, respectively, was confirmed. Better understanding of these NAC gene family and identifying the particular function of the specific NACs is most important in agriculture. A detailed regulatory mechanism of these potential stress-related individual NAC genes and their possible interactions provides an opportunity to understand the molecular basis of drought tolerance in these legume crops, that could allow improved varieties to be developed with ample accuracy. Therefore, these genes are valuable resources for further gene function validation and their subsequent use in genetic engineering and molecular breeding for addressing drought stress in legume crops.

It is crucial to mention here that the present study provides very detailed analysis of the identified NAC genes in pigeonpea and chickpea as compared to the previous reports by Satheesh et al. [54] and Ha et al. [53], respectively. We have carried out rigorous motif analysis, promoter analysis, and protein-protein interaction studies of putative stress-related NAC proteins which were lacked in previous reports and therefore, provides much deeper understanding of mechanisms involved in drought stress tolerance in chickpea. Similarly, the findings of Satheesh et al. [54] involved only in silico analysis, whereas NAC transcription factors have not been systematically researched in groundnut, till date. Hence, the present work generates large data sets that further can be used as base for more sophisticated and targeted studies in future. It managed to put attention on the importance of further understanding the potential of legume NAC genes (*Ca_NAC*, *Cc_NAC*, and *Ah_NAC*), for the purpose of improving abiotic stress tolerance in general.

## Conclusions

It is well known that NAC genes play important roles during developmental and abiotic stress responses. Though, few NAC genes have been identified in chickpea and pigeonpea that are involved in drought response. Therefore, to find such notable genes in chickpea, pigeonpea and groundnut was not the only aim of this study. It further aimed to obtain insight into the transcription patterns and putative functions of NAC genes in these legumes. Based on the genome sequence, we have comprehensively identified NAC genes in three SAT legumes viz., chickpea, pigeonpea, and groundnut. A non-redundant set of 72, 96, and 166 NAC genes were detected in chickpea, pigeonpea, and groundnut, respectively. Detailed analyses revealed phylogenetic association, conserved domains, gene structure, transmembrane helices, promoter analysis, gene interaction networks, and expression profiles of NAC genes among these three legumes. Based on data gathered during this investigation, we could identify 21 potential NAC genes for drought tolerance in legumes. This study has furthered our knowledge of legume NAC genes and provided insight into their functions. Furthermore, expression analyses for putative NAC genes during developmental stages and drought exposure confirmed our findings, and have built a robust framework for researchers to select candidates to engineer chickpea, pigeonpea, and groundnut cultivars for enhanced tolerance against drought stress.

## Methods

### Plant material and drought stress imposition

Two contrasting drought-responsive genotypes each for chickpea - ICC 4958 (tolerant) and ICC 1882 (sensitive), pigeonpea - ICPL 227 (more tolerant) and ICPL 151 (less tolerant), and groundnut - CSMG 84–1 (tolerant) and ICGS 76 (sensitive) were selected for the study. The seeds of selected cultivated genotypes (approx. 10–12 seeds) were procured from the Chickpea Breeding unit, Research Program – Asia of the International Crops Research Institute for the Semi-Arid Tropics (ICRISAT), Patancheru, India. Seeds were thoroughly rinsed with distilled water and germinated on moist filter paper. Germinating seedlings were then transferred to pots filled with autoclaved soil under controlled glasshouse conditions after the emergence of radicle and cotyledonary leaves. Drought stress was imposed on 30-day-old chickpea, pigeonpea, and groundnut plants using polyethylene glycol (PEG) induced treatment (20% PEG 8000). Root tissues were collected six days after PEG treatment and stored in − 80 °C until RNA isolation.

### Identification and data analyses of NAC family genes/proteins in chickpea, pigeonpea, and groundnut

Previously-identified NAC protein sequences from other plant species such as *Arabidopsis*, *Medicago, Lotus, Glycine max,* etc., were searched against the predicted gene models of three legumes (chickpea, pigeonpea, and groundnut) using blastp program at a cutoff threshold E-value of ≤1E-05. In addition to this, the HMM profile of the NAC family was extracted from the Pfam database [77], and NAC HMM profile was scanned against the predicted gene models of legumes under study for target hits with the NAC domain by HMMER v2.1.1 [78]. The identified proteins/genes were further confirmed for the presence of NAC domain using SMART and Pfam searches. The physio-chemical properties of the identified NAC proteins, such as the number of amino acids in the open reading frame (ORF), molecular weight (MW), isoelectric point (pI), and length of each gene was determined using ExPASy (http://www.expasy.ch/tools/pi_tool.html). Softberry (http://linux1.softberry.com/) was used to predict subcellular localization of the identified NAC family proteins using ProtComp (Program for predicting protein sub-cellular location). Subcellular localization predictions were based on significant similarity in Potential Location database by DBSCAN (database homology search program similar to BLAST). MapChart 2.32 software (https://www.wur.nl/en/show/Mapchart.htm) was used to represent the chromosomal distribution of these identified NAC genes.

### Transmembrane domains prediction and orthologs distribution

TMHMM v2.0 (http://www.cbs.dtu.dk/services/TMHMM/) was used to determine the transmembrane helices. To identify orthologs, chickpea, pigeonpea, and groundnut NAC proteins were searched against the whole set of *Medicago* and soybean proteins using blastp program applying a threshold E-value of 1E− 10, 80% similarity, and 80% query coverage. Further, circos [34] was used to represent these orthologous relationships.

### Phylogenetic analysis and identification of putative stress-responsive NAC genes

MEGA (V7.0) software (http://www.megasoftware.net/) was used to perform phylogenetic relationships. Neighbor-Joining (NJ) method with bootstrap values more than 30% was used to construct unrooted phylogenetic tree/s. Further, for identifying putative stress-responsive NAC genes, a total of 43 abiotic stress-responsive NAC protein sequences (from *Arabidopsis*, *Oryza sativa*, *Medicago truncatula* and *Glycine max*) were included along with the NAC protein sequences of legume crops, and sequence alignments were performed.

### Conserved motifs and gene structure analysis of putative stress-responsive NAC genes

The motif prediction was done at different motif widths using MEME standalone version 5.0.2 [79]. MEME for conserved motifs with parameters like 20 number of motifs, 10–50 motif width, and 2–72 motif sites (2–96 for pigeonpea and 2–166 for groundnut), with E-value threshold of 0.05 were used. GSDS 2.0 (Gene Structure Display Server) was used to visualize the exon/intron organization of the putatively identified stress-responsive NAC genes [80].

### Prediction of *cis*-acting regulatory elements (CAREs) in putative stress-responsive NACs

The upstream promoter sequences of identified stress-responsive NAC genes (1500-bp sequences upstream of the translation initiation codon) were analyzed for the presence of putative CAREs using the PlantCARE (Plant *Cis*-Acting Regulatory Elements) database [81]. *Cis*-elements with matrix score above five were considered.

### STRING analysis for protein-protein interaction studies

Web-based STRING (Search Tool for the Retrieval of Interacting Genes/Proteins) database version 11.0 (https://string-db.org/) was used to carry out protein-protein network studies. Stress-related chickpea, pigeonpea, and groundnut NAC protein sequences were searched against *Arabidopsis thaliana* proteins for the identification of best corresponding hits. Thus, the resulting hits were used for protein-protein interaction analysis.

### *In-Silico* expression analysis of NAC genes in chickpea, pigeonpea, and groundnut

Expression data for putative stress-responsive NACs in different tissues collected at various developmental stages – including germination, seedling, vegetative, reproductive and senescence – was retrieved from *Cicer arietinum* (chickpea) Gene Expression Atlas (CaGEA) [35], *Cajanus cajan* (pigeonpea) gene expression atlas (CcGEA) [36], and *Arachis hypogaea* (groundnut) Gene Expression Atlas [37]. Expression patterns of these NAC genes were analyzed and represented as heat map viewed in MeV tool version 4.9.0 (https://sourceforge.net/projects/mev-tm4/).

### RNA isolation

Total RNA was isolated using the Nucleospin RNA plant kit (Macherey-Nagel, Duren, Germany) as per the manufacturer’s instructions from root tissues collected from both stressed and well-watered plants of contrasting drought-responsive genotypes of the three legumes. First-strand cDNA synthesis was performed using SuperScript®III RT enzyme (Invitrogen, CA, USA).

### Quantitative real-time PCR (qRT-PCR) analysis

Quantitative real-time PCR (qRT-PCR) was performed on Applied Biosystems 7500 Real Time PCR System using SYBR Green-chemistry (Applied Biosystems, USA). The *glyceral-dehyde-3-phosphate dehydrogenase*, *actin*, and *alcohol dehydrogenase* genes were used as an endogenous control for chickpea, pigeonpea, and groundnut, respectively. The reactions were performed with three biological and two technical replicates. 2^-△△CT^ method was used to calculate relative expression levels [82]. Specific primers for qRT-PCR were designed using PrimerQuest tool (https://eu.idtdna.com/scitools/Applications/RealTimePCR/Default.aspx).

## Supplementary Information

**Additional file 1: Table S1** Putative NAC family genes identified in chickpea (72), pigeonpea (96) and groundnut (166). **Table S2** Details of well-known NAC genes from model and crop plants. **Table S3** Summary of conserved motifs for chickpea, pigeonpea, and groundnut NAC genes. **Table S4** Detailed analysis of conserved motifs for chickpea, pigeonpea and groundnut NAC genes. **Table S5** Predicted promoter elements for stress-responsive chickpea, pigeonpea and groundnut NAC genes. **Table S6** Possible matches of chickpea, pigeonpea, and groundnut stress-responsive NAC protein sequences with *Arabidopsis* proteins. **Table S7** STRING analysis for protein-protein interaction analysis of stress-responsive NACs from SAT legumes. **Table S8** Corresponding transcript ids of groundnut with *Arachis hypogaea* gene expression atlas (AhGEA). **Table S9** List of NAC-specific primers used for qRT-PCR analysis in selected legumes.

**Additional file 2: Fig. S1** Prediction of stress-responsive *Ca_NAC* genes based on phylogenetic analysis using MEGA7.0. A total of 107 protein sequences were used which included 72 from chickpea and 43 well-known stress-responsive NAC genes from *Arabidopsis thaliana*, *Oryza sativa, Medicago truncatula* and *Glycine max*. Bootstrap values are displayed next to the branch nodes. **Fig. S2** Prediction of stress-responsive *CcL_NAC* genes based on phylogenetic analysis using MEGA7.0. A total of 139 protein sequences were used which included 96 from pigeonpea and 43 well-known stress-related NAC genes from *Arabidopsis thaliana*, *Oryza sativa, Medicago truncatula* and *Glycine max*. Bootstrap values are displayed next to the branch nodes. **Fig. S3** Prediction of stress-responsive *Ah_NAC* genes based on phylogenetic analysis using MEGA7.0. A total of 209 protein sequences were used which included 166 from groundnut and 43 well-known stress-related NAC genes from *Arabidopsis thaliana*, *Oryza sativa, Medicago truncatula* and *Glycine max*. Bootstrap values are displayed next to the branch nodes. **Fig. S4** Representation of protein-protein interactions among predicted stress-responsive chickpea, pigeonpea, and groundnut proteins using STRING database v11.0.

## Data Availability

All data generated or analyzed in the study are included in this article and its supplementary information files. The gene expression atlas data sets from tissue specific RNA-seq analysis used in this study was publicly available at- *Cicer arietinum* (10.1111/pce.13210) [35], *Cajanus cajan* (10.1093/jxb/erx010) [36] and *Arachis hypogea* (10.1111/pbi.13374) [37].

## Abbreviations

CAREs: *Cis*-Acting Regulatory Elements
HMM: Hidden Markov Model
MW: Molecular Weight
NJ: Neighbor-Joining
ORF: Open Reading Frame
SAT: Semi-Arid Tropics
STRE: Stress-Responsive Element
STRING: Search Tool for the Retrieval of Interacting Genes
TFs: Transcription Factors
