# Supplementary figures and images for: Comprehensive analysis and identification of drought-responsive candidate NAC genes in three semi-arid tropics (SAT) legume crops
Source: BMC Genomics. 2021 Apr 21;22:289. doi: 10.1186/s12864-021-07602-5 (PMC8059324; doi:10.1186/s12864-021-07602-5)

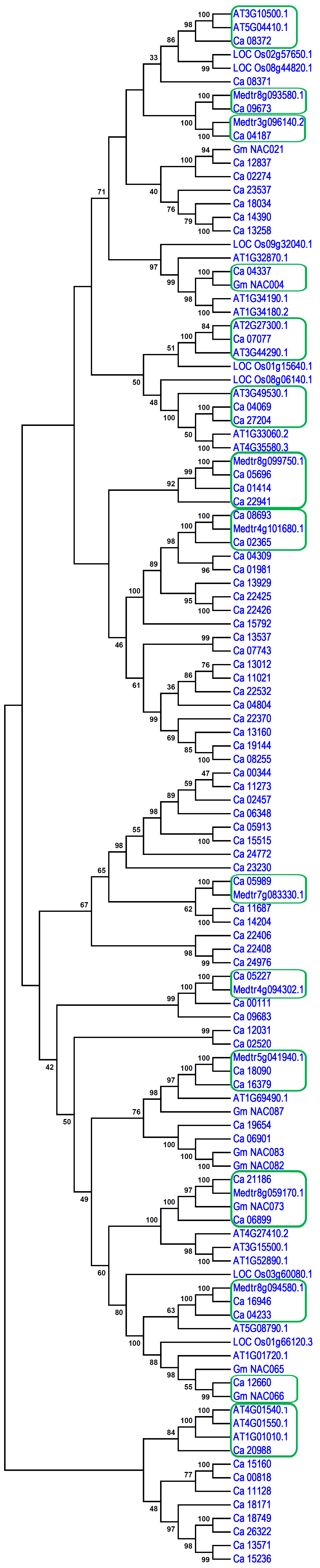

Supplement: Supplementary file 2 — Additional file 2: Fig. S1 Prediction of stress-responsive Ca_NAC genes based on phylogenetic analysis using MEGA7.0. A total of 107 protein sequences were used which included 72 from chickpea and 43 well-known stress-responsive NAC genes from Arabidopsis thaliana, Oryza sativa, Medicago truncatula and Glycine max. Bootstrap values are displayed next to the branch nodes. Fig. S2 Prediction of stress-responsive CcL_NAC genes based on phylogenetic analysis using MEGA7.0. A total of 139 protein sequences were used which included 96 from pigeonpea and 43 well-known stress-related NAC genes from Arabidopsis thaliana, Oryza sativa, Medicago truncatula and Glycine max. Bootstrap values are displayed next to the branch nodes. Fig. S3 Prediction of stress-responsive Ah_NAC genes based on phylogenetic analysis using MEGA7.0. A total of 209 protein sequences were used which included 166 from groundnut and 43 well-known stress-related NAC genes from Arabidopsis thaliana, Oryza sativa, Medicago truncatula and Glycine max. Bootstrap values are displayed next to the branch nodes. Fig. S4 Representation of protein-protein interactions among predicted stress-responsive chickpea, pigeonpea, and groundnut proteins using STRING database v11.0. [file 12864_2021_7602_MOESM2_ESM.zip › Figure S1.tif]

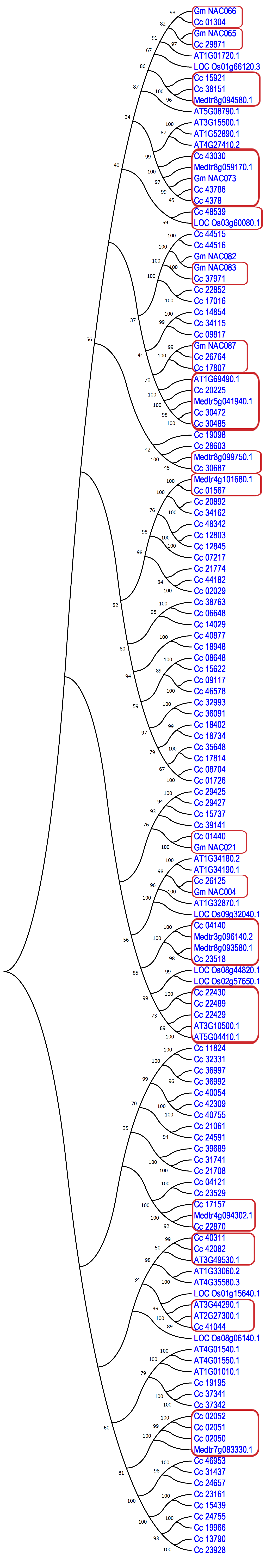

Supplement: Supplementary file 2 — Additional file 2: Fig. S1 Prediction of stress-responsive Ca_NAC genes based on phylogenetic analysis using MEGA7.0. A total of 107 protein sequences were used which included 72 from chickpea and 43 well-known stress-responsive NAC genes from Arabidopsis thaliana, Oryza sativa, Medicago truncatula and Glycine max. Bootstrap values are displayed next to the branch nodes. Fig. S2 Prediction of stress-responsive CcL_NAC genes based on phylogenetic analysis using MEGA7.0. A total of 139 protein sequences were used which included 96 from pigeonpea and 43 well-known stress-related NAC genes from Arabidopsis thaliana, Oryza sativa, Medicago truncatula and Glycine max. Bootstrap values are displayed next to the branch nodes. Fig. S3 Prediction of stress-responsive Ah_NAC genes based on phylogenetic analysis using MEGA7.0. A total of 209 protein sequences were used which included 166 from groundnut and 43 well-known stress-related NAC genes from Arabidopsis thaliana, Oryza sativa, Medicago truncatula and Glycine max. Bootstrap values are displayed next to the branch nodes. Fig. S4 Representation of protein-protein interactions among predicted stress-responsive chickpea, pigeonpea, and groundnut proteins using STRING database v11.0. [file 12864_2021_7602_MOESM2_ESM.zip › Figure S2.tif]

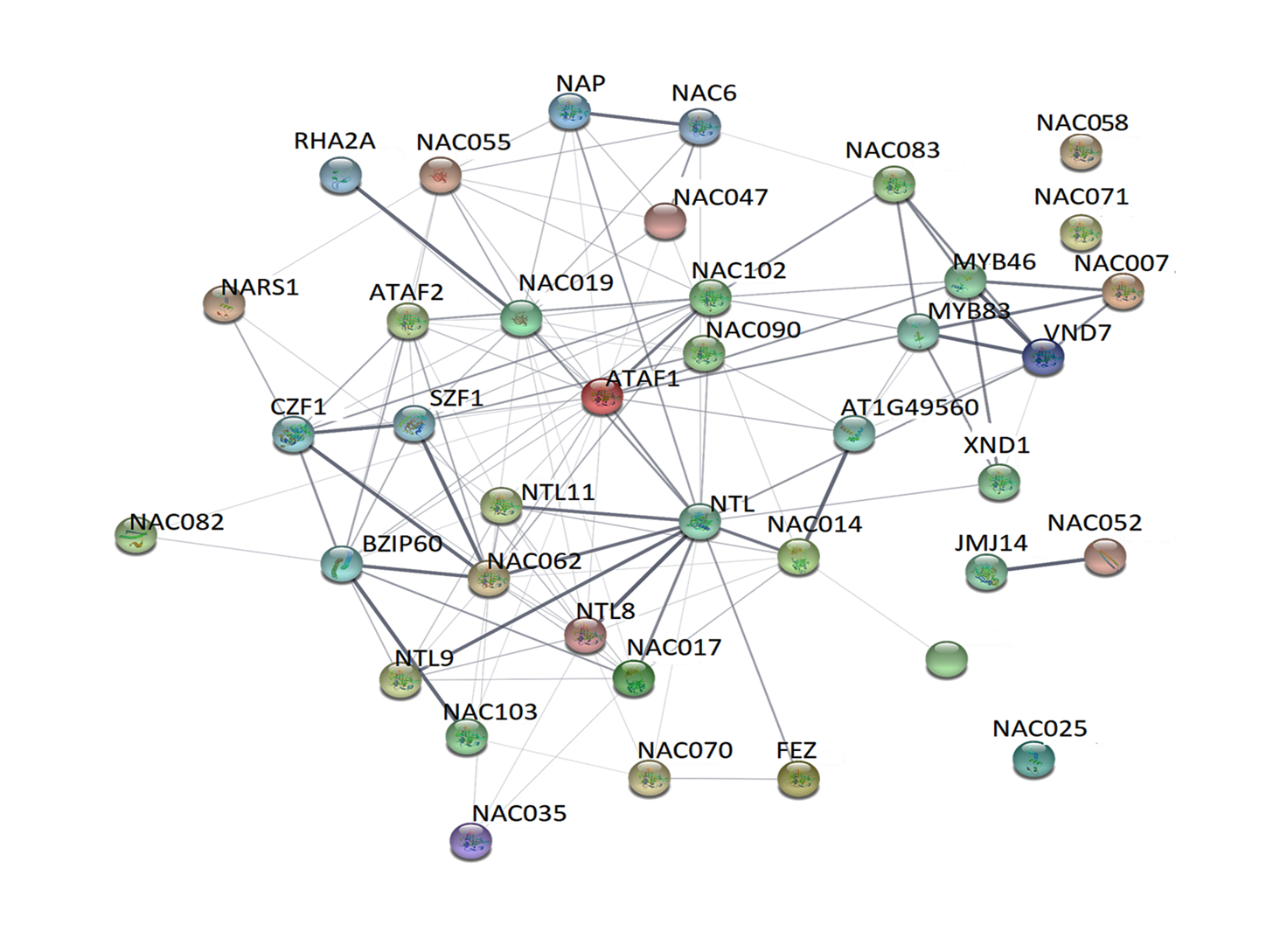

Supplement: Supplementary file 2 — Additional file 2: Fig. S1 Prediction of stress-responsive Ca_NAC genes based on phylogenetic analysis using MEGA7.0. A total of 107 protein sequences were used which included 72 from chickpea and 43 well-known stress-responsive NAC genes from Arabidopsis thaliana, Oryza sativa, Medicago truncatula and Glycine max. Bootstrap values are displayed next to the branch nodes. Fig. S2 Prediction of stress-responsive CcL_NAC genes based on phylogenetic analysis using MEGA7.0. A total of 139 protein sequences were used which included 96 from pigeonpea and 43 well-known stress-related NAC genes from Arabidopsis thaliana, Oryza sativa, Medicago truncatula and Glycine max. Bootstrap values are displayed next to the branch nodes. Fig. S3 Prediction of stress-responsive Ah_NAC genes based on phylogenetic analysis using MEGA7.0. A total of 209 protein sequences were used which included 166 from groundnut and 43 well-known stress-related NAC genes from Arabidopsis thaliana, Oryza sativa, Medicago truncatula and Glycine max. Bootstrap values are displayed next to the branch nodes. Fig. S4 Representation of protein-protein interactions among predicted stress-responsive chickpea, pigeonpea, and groundnut proteins using STRING database v11.0. [file 12864_2021_7602_MOESM2_ESM.zip › Figure S4.tif]
